# Supplementary material for: Genetic associations with temporal shifts in obesity and severe obesity during the obesity epidemic in Norway: A longitudinal population-based cohort (the HUNT Study)
Source: PLoS Med. 2020 Dec 14;17(12):e1003452. doi: 10.1371/journal.pmed.1003452 (PMC7735641; doi:10.1371/journal.pmed.1003452)
Supplement: S1 Protocol — (DOCX) [file pmed.1003452.s001.docx]

# Prospective Protocol for Maria Brandkvist

Head supervisor: Gunnhild Åberge Vie

Co-supervisors: Johan Håkon Bjørngaard, Rønnaug Ødegård

# Article

Over that last decades, there has been a quest to find the missing heritability of obesity. Twin and adoptions studies indicate a strong genetic contribution at the individual level with heritability estimates for obesity between 40 and 60%. ^1, 2^ Although relying on family observations, this rough estimate encompasses both the additive effect of genes and multiplicative effects such as gene-environment interplay. ^3^ In contrast with these estimates, genome-wide association studies (GWAS) have identified genetic variants that explain a mere fraction of the additive effect of genes that accounts for 2-5% of variation in BMI.

Recently, a genome wide polygenic score (GPS) has been developed for BMI that combines ‘all available common variants into a single quantitative measure of inherited susceptibility’. ^4^ Unlike the genetic risk score (GRS) based on 97 genetic variants reaching genome-wide significance for BMI^5^, the GPS encompasses over two million genetic variants associated with BMI. By using most of the information in our genome, we can explain a larger variation of BMI in the population. This powerful new tool retrieves some of the missing heritability of obesity by increasing variance from 2-5% in GWAS to 9% in GPS. This translates to a 13-kg weight gradient in today’s middle-aged adults solely due to genetic differences.^4^ For the first time, any ‘reasonably sensitive observer’ can appreciate the genetic contribution to obesity.^6^

The GPS has room for refinement and accounts for only the additive effect of common gene variants. However, it can be used as a tool for exploring gene-environment interactions, ‘which are known to be important for genetic effects on’ obesity.^7^ Previous studies have suggested that genetic variants known to predict BMI had larger effects after the onset of the obesity epidemic than before, however, they are limited by self-reported BMI, short follow-up time, a selected older population ^8, 9^ and by weaker genetic instruments. (Ref .Brandkvist/Vie in print BMJ)

Using a comprehensive dataset with the largest sample size and range of ages and years to date, we aim to study the absolute risk for obesity and heavier body weight for individuals in the top decile of the GPS score. We will investigate if recent secular trends affected this risk among the most genetically predisposed. We also aim to demonstrate the stability of the GPS in an external sample and before and after the obesity epidemic.

## Method:

*Study population*

The study is based on data from participants in the Nord-Trøndelag Health Study (HUNT, 1984-2008) linked to previous height and weight measurements for the same individuals in the Tuberculosis Screening Program (1963-75). We will include 118,959 HUNT participants aged 13-80 with valid height and weight measurements of whom 67,305 had genetic data available.

*Weight and Obesity*

The main outcomes will be weight and the prevalence of obesity. Body mass index (BMI) will be a secondary outcome with corresponding results shown in the supplementary materials. We chose to focus on weight in relation to men and women of average stature as this outcome is comprehendible for the general reader. Participants were measured wearing light clothes and without shoes. Weight was measured to the nearest half kilogram and height was measured to the nearest centimeter^10^. We will calculated BMI as weight in kilograms per meter squared*.* The World Health Organization defines overweight as BMI greater than or equal to 25 and obesity as BMI greater than or equal to 30 ^11^. We will calculate a BMI z-score for participants younger than 18 years of age as BMI strongly relates to longitudinal growth. Here we will use the International Obesity Task Force (IOTF) reference to adjust for age and gender^12^.

*Genotyping, derivation and validation of the Genome-Wide Polygenic Score*

For adult participants in HUNT2 and HUNT3, genotyping was performed with one of three different Illumina HumanCoreExome arrays (HumanCoreExome12v1.0, HumanCoreExome12 v1.1, and UM HUNT Biobank v1.0).^13^

We will use the genome-wide polygenic score (GPS) for BMI recently derived, validated and tested by Khera et al. This GPS provides a ‘single quantitative measure of inherited susceptibility’ by combining all available common variants (minor allele frequency >=1%) for BMI.^4^

The GPS was derived using a computational algorithm for 2.1 million genetic variants identified to be associated with BMI in over 300,000 individuals in the Giant Investigation of Anthropometric Traits consortium (GIANT)^4, 5^. Please see Khera et al. for more detail on derivation and validation of the GPS.^4^ We will use weights from Khera et al. that were estimated using the UK Biobank. We will divide the study population into ten equal sized groups.

*Statistical Analysis*

We will analyze longitudinal trajectories in obesity (BMI >30 kg/m^2^ as a dichotomous variable) using linear mixed models with observations clustered within individuals, and with a random slope for age. We will adjust for age using linear splines. The analyses will be performed separately for men and women. Based on this model, we will graph the estimated prevalence of obesity for the highest compared to lowest tenth of genetic susceptibility for chosen ages at each decade for men and women. Using a linear model on a dichotomous outcome gives us a linear probability model. The results will be easily interpretable, but we risk having confidence intervals including impossible values of probabilities below null. We will use a similar model to analyze longitudinal trajectories in weight (kg) in relation to men and women of average height.

We will perform several sensitivity analyses. First, we will analyze the associations between genetic risk, age and time using generalized estimation equation (GEE). This will allow us to use a logistic model. By use of postestimation commands (margins), we can predict the probability of obesity in the population for different groups. Second, we will assess the probability of having genetic data available (i.e. participating in HUNT2 or HUNT3) based on the obesity status of participants in TBC-screening. An association will indicate a possible selection bias, and will be handled by restricting the sample to younger cohorts. Third, we will assess the association between GPS and the natural logarithm of BMI. We will do this to approximate the relative difference in BMI rather than the absolute difference in BMI between the top and bottom tenth of genetic predisposition.

# Ethics

We obtained ethical clearance from the Regional Committees for Medical and Health Research Ethics for this project through the main project “Burden of obesity in Norway”. We recently extended this ethical clearance for the analyses using all genetic variants associated with BMI and educational attainment. The project is approved by the data inspectorate and applications for linkages are approved by the data owners. The project is based on observational data already collected. There is no intervention; there is therefore no known risk for the participants. The collection of large datasets including genetic information nonetheless requires scrutiny in handling data. Furthermore, scrutiny in presenting research results is necessary to avoid adverse outcomes and misinterpretation.

Patient and public involvement

No patients were involved in setting the research question or the outcome measures, nor were they involved in the design or implementation of both studies. As the studies used previously collected data, we did not ask patients or the public to assess the burden of participation. We will seek involvement from a patient organisation in the development of an appropriate method of dissemination.

1. Rokholm B, Silventoinen K, Tynelius P, Gamborg M, Sorensen TI, Rasmussen F. Increasing genetic variance of body mass index during the Swedish obesity epidemic. PLoS One. 2011;6(11):e27135.

2. Silventoinen K, Jelenkovic A, Sund R, Yokoyama Y, Hur YM, Cozen W, et al. Differences in genetic and environmental variation in adult BMI by sex, age, time period, and region: an individual-based pooled analysis of 40 twin cohorts. Am J Clin Nutr. 2017;106(2):457-66.

3. Plomin R, von Stumm S. The new genetics of intelligence. Nature reviews Genetics. 2018;19(3):148-59.

4. Khera AV, Chaffin M, Wade KH, Zahid S, Brancale J, Xia R, et al. Polygenic Prediction of Weight and Obesity Trajectories from Birth to Adulthood. Cell. 2019;177(3):587-96.e9.

5. Locke AE, Kahali B, Berndt SI, Justice AE, Pers TH, Day FR, et al. Genetic studies of body mass index yield new insights for obesity biology. Nature. 2015;518(7538):197-206.

6. Plomin R. Blueprint. Great Britain: Penguin Random House UK; 2018.

7. Lee JJ, Wedow R, Okbay A, Kong E, Maghzian O, Zacher M, et al. Gene discovery and polygenic prediction from a genome-wide association study of educational attainment in 1.1 million individuals. Nat Genet. 2018;50(8):1112-21.

8. Walter S, Mejia-Guevara I, Estrada K, Liu SY, Glymour MM. Association of a Genetic Risk Score With Body Mass Index Across Different Birth Cohorts. JAMA. 2016;316(1):63-9.

9. Goodarzi MO. Genetics of obesity: what genetic association studies have taught us about the biology of obesity and its complications. The lancet Diabetes & endocrinology. 2018;6(3):223-36.

10. Krokstad S, Langhammer A, Hveem K, Holmen TL, Midthjell K, Stene TR, et al. Cohort Profile: the HUNT Study, Norway. Int J Epidemiol. 2013;42(4):968-77.

11. Obesity and Overweight: World Health Organization; [Available from: <http://www.who.int/en/news-room/fact-sheets/detail/obesity-and-overweight> (2018-08-02).

12. Cole TJ, Lobstein T. Extended international (IOTF) body mass index cut-offs for thinness, overweight and obesity. Pediatr Obes. 2012;7.

13. Nielsen JB, Thorolfsdottir RB, Fritsche LG, Zhou W, Skov MW, Graham SE, et al. Genome-wide association study of 1 million people identifies 111 loci for atrial fibrillation. bioRxiv. 2018.
