# Supplementary material for: Genetic associations with temporal shifts in obesity and severe obesity during the obesity epidemic in Norway: A longitudinal population-based cohort (the HUNT Study)
Source: PLoS Med. 2020 Dec 14;17(12):e1003452. doi: 10.1371/journal.pmed.1003452 (PMC7735641; doi:10.1371/journal.pmed.1003452)
Supplement: S1 Text — (DOCX) [file pmed.1003452.s002.docx]

Modifications made to the prospective protocol and rationale

The analytical approach used in the paper ‘Genetic inequality in obesity and severe obesity during the obesity epidemic: longitudinal findings from the HUNT Study verified by sibling design’ is slightly modified from the prospective protocol. All modifications were made prior to commencing the analyses and in agreement with all co-authors. The following is the rationale for the modifications made to the prospective protocol for each respective section.

*Study population:*

We were able to include BMI data as recent as 2019 as data from the HUNT 4 Survey became available. We chose include the new data as it expands our dates of assessment and conveys periods with both increasing and stabilizing prevalence of obesity.

*Weight and obesity:*

We changed our main outcome from weight to height adjusted BMI. We chose this modification as height adjusted BMI can easily be translated to obesity and severe obesity. Adjusting BMI for height may seem counterintuitive as the BMI formula incorporates height. Nevertheless, it is still debated whether the BMI formula accounts for weight differences amongst people of different heights appropriately. (1) Although BMI is not especially correlated with height, we chose to adjust BMI for height in order to account for any effect of the six centimeters height increase in the population since the 1960s. (2) We later redid the analyses with BMI and found similar results.

Also, severe obesity was not described in the original protocol. We chose to include severe obesity as this category is strongly associated with physical and psychiatric multimorbidity.

*Statistical analyses:*

In the statistical analyses, we did not use a linear model for dichotomous outcomes. Instead we modelled the association of GPS with obesity and severe obesity using generalized estimating equations. Analyses using generalized estimation equations (GEE) were performed with obesity and severe obesity in the main analyses rather than in supplementary analyses.

Analyses were not performed separately for men and women as we found it simpler to use an interaction term for sex. In the protocol, we also wrote that we would use a similar model to the main analyses *‘to analyze longitudinal trajectories in weight (kg) in relation to men and women of average height.’* This was not done as we chose to focus on height adjusted BMI.

In the sensitivity analyses, we assessed the probability of having genetic data available (i.e. participating in HUNT2 or HUNT3) based on the obesity status of participants in TBC-screening. However, we chose not to focus on these results nor include them in the manuscript. Hence, we did not handle any selection bias by restricting the sample to younger cohorts.

Originally, we did not assess the association between GPS and the natural logarithm of BMI. Additionally, we included plots with Kernel density estimates for the BMI distribution. This was done in response to comments from the reviewers.

1. Michels KB, Greenland S, Rosner BA. Does body mass index adequately capture the relation of body composition and body size to health outcomes? Am J Epidemiol. 1998;147(2):167-72.

2. Roser M, Appel C, Ritchie H. Human Height 2013 [updated May 20192019-09-03]. Available from: <https://ourworldindata.org/human-height>.
