# Supplementary material for: Genetic associations with temporal shifts in obesity and severe obesity during the obesity epidemic in Norway: A longitudinal population-based cohort (the HUNT Study)
Source: PLoS Med. 2020 Dec 14;17(12):e1003452. doi: 10.1371/journal.pmed.1003452 (PMC7735641; doi:10.1371/journal.pmed.1003452)
Supplement: S2 Text — (DOCX) [file pmed.1003452.s003.docx]

**Supporting Information**

**Contents**

**Fig A Linearity of the association between the GPS for BMI and BMI.** Linearity of the association between the GPS for BMI and BMI for 25, 45, and 65 year old men and women.

**Fig B Estimated prevalence of obesity by top and bottom tenth of genome-wide polygenic score for ages 15-65**. Estimated prevalence (%, with 95% confidence interval) of obesity (BMI ≥ 30 kg/m^2^) by top (most susceptible, circle) and bottom tenth (least susceptible, x) of genome-wide polygenic score by age and time point for 31,717 men and 35,393 women who participated in the HUNT Study, Norway. *Youngest observed age in 2017-19 was 28.6 years.

**Fig C Estimated prevalence of severe obesity by top and bottom tenth of genome-wide polygenic score for ages 15-65.** Estimated prevalence (%, with 95% confidence interval) of severe obesity (BMI ≥ 35 kg/m^2^) by top (most susceptible, circle) and bottom tenth (least susceptible, x) of genome-wide polygenic score by age and time point for 31,717 men and 35,393 women who participated in the HUNT Study, Norway *Youngest observed age in 2017-19 was 28.6 years.

**Fig D Cumulative prevalence of different weight categories.** Cumulative prevalence of underweight, normal weight, overweight, obesity and severe obesity for the entire study population by time point.

**Fig E Kernel plot of BMI distribution for the entire study sample by time.** Kernel plot of BMI distribution for the entire study sample by time of measurement among 67,110 participants in the HUNT Study.

**Fig F Kernel plot of BMI distribution by time, age and GPS.** Kernel plot of BMI distribution by time of measurement, age and categories of genome-wide polygenic score distribution (GPS) among 65,432 participants age 20-60 in the HUNT Study.

**Fig G Estimated height adjusted BMI by top and bottom tenth of GPS.** Estimated height-adjusted BMI (with 95% confidence interval) by top (most susceptible, circle) and bottom tenth (least susceptible, x) of genome-wide polygenic score by age and time point for 31,717 men and 35,393 women who participated in the HUNT Study, Norway. *Youngest observed age in 2017-19 was 28.6 years.

**Fig** **H** **Estimated BMI by top and bottom tenth of GPS.** Estimated BMI (with 95% confidence interval) by top (most susceptible, circle) and bottom tenth (least susceptible, x) of genome-wide polygenic score by age and time point for 31,717 men and 35,393 women who participated in the HUNT Study, Norway. *Youngest observed age in 2017-19 was 28.6 years.

**Fig I Estimated association between genetic risk and BMI within and between siblings.** Estimated association between one standard unit higher GPS for height-adjusted BMI within and between siblings by year. Based on 29,585 individuals composing 11,857 sibling groups within participants in the HUNT Study.

**Fig J Estimated BMI using data from first degree relatives by top and bottom tenth of GPS.** Estimated height-adjusted BMI using data from first degree relatives by top (most susceptible, circle) and bottom tenth (least susceptible, x) of genome-wide polygenic score by age and time point for 31,717 men and 35,393 women who participated in the HUNT Study, Norway.

**Fig K Estimated obesity prevalence using data from first degree relatives by top and bottom tenth of GPS.** Estimated prevalence of obesity using data from first degree relatives by top (most susceptible, circle) and bottom tenth (least susceptible, x) of genome-wide polygenic score by age and time point for 31,717 men and 35,393 women who participated in the HUNT Study, Norway.

**Fig L Estimated prevalence of severe obesity using data from first degree relatives by top and bottom tenth of GPS.** Estimated prevalence of severe obesity using data from first degree relatives by top (most susceptible, circle) and bottom tenth (least susceptible, x) of genome-wide polygenic score by age and time point for 31,717 men and 35,393 women who participated in the HUNT Study, Norway.

**Table A The distribution of participants according to both the** **genome-wide polygenic score (GPS) and genetic risk score (GRS).**

**Table B Mean BMI for the bottom and top tenth of genetic predisposition according to the genome-wide polygenic score (GPS) and the genetic risk score (GRS).**

**Table C Estimated difference BMI between the tenths with highest and lowest genetic susceptibility at various time points.**

**Table D Estimated difference in height adjusted BMI over time for the tenths with the highest and lowest genetic susceptibility for men and women combined.**

**Table E Estimated difference in BMI over time for the tenths with the highest and lowest genetic susceptibility for men and women combined.**

**Table F Estimated difference in BMI between the tenths with the highest and lowest genetic susceptibility over time for all ages.**

**Table G Estimates from sibling analysis with BMI. Body mass index (BMI). Difference is the between sibship coefficient minus the within sibship coefficient.**

**Table H Estimates from sibling analysis with obesity. Odds ratio (OR). Difference is the between sibship coefficient minus the within sibship coefficient.**

**Table I Estimated difference in natural logarithm of BMI between the tenths with the highest and lowest genetic susceptibility over time.**


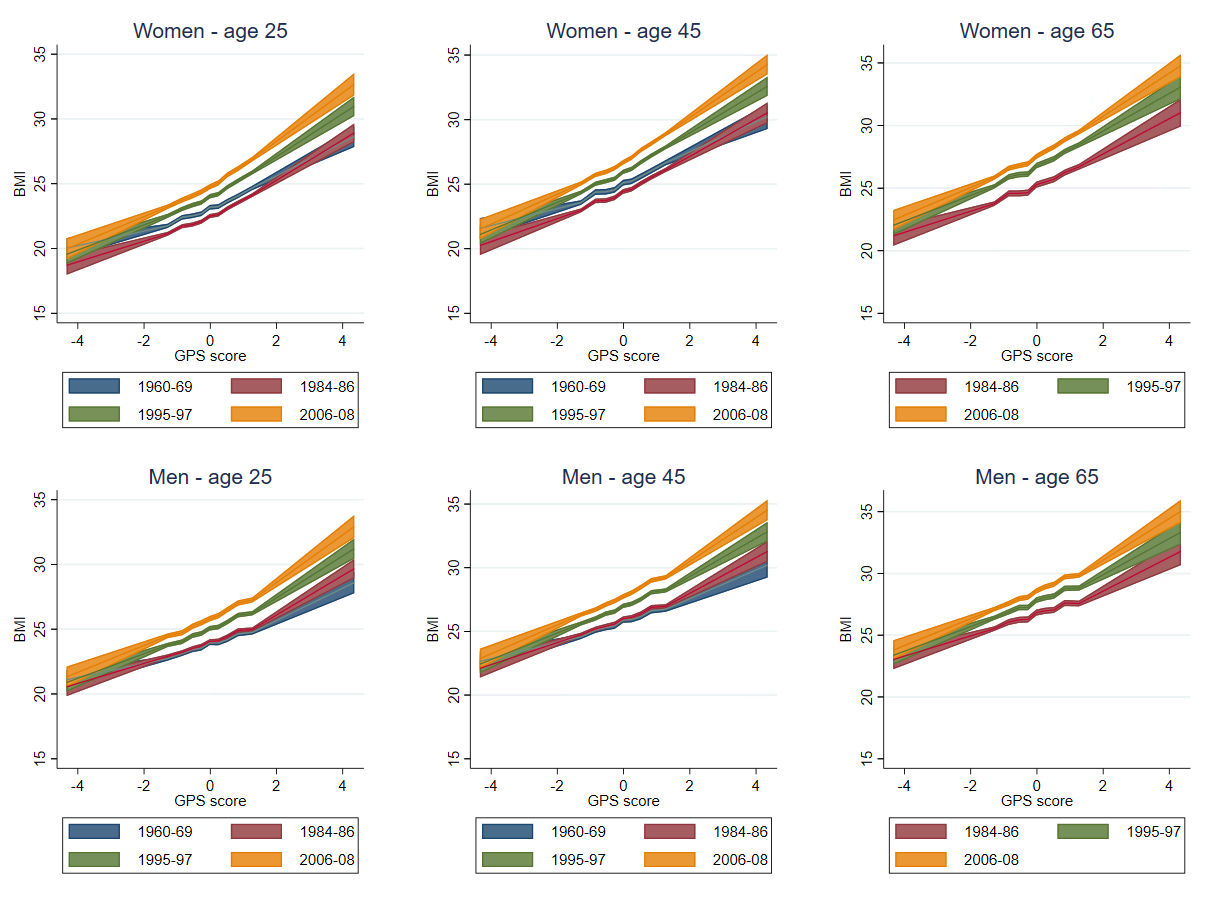


Fig A Linearity of the association between GPS for BMI and BMI for 25, 45, and 65 year old men and women.


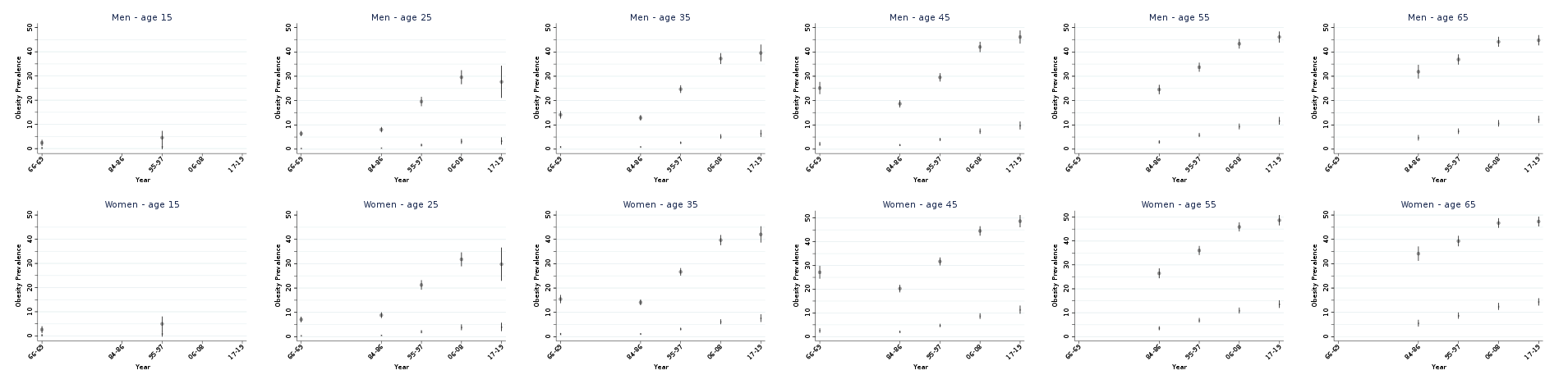


Fig B Estimated prevalence (%, with 95% confidence interval) of obesity (BMI ≥ 30 kg/m^2^) by top (most susceptible, circle) and bottom tenth (least susceptible, x) of genome-wide polygenic score by age and time point for 31,717 men and 35,393 women who participated in the HUNT Study, Norway. *Youngest observed age in 2017-19 was 28.6 years.


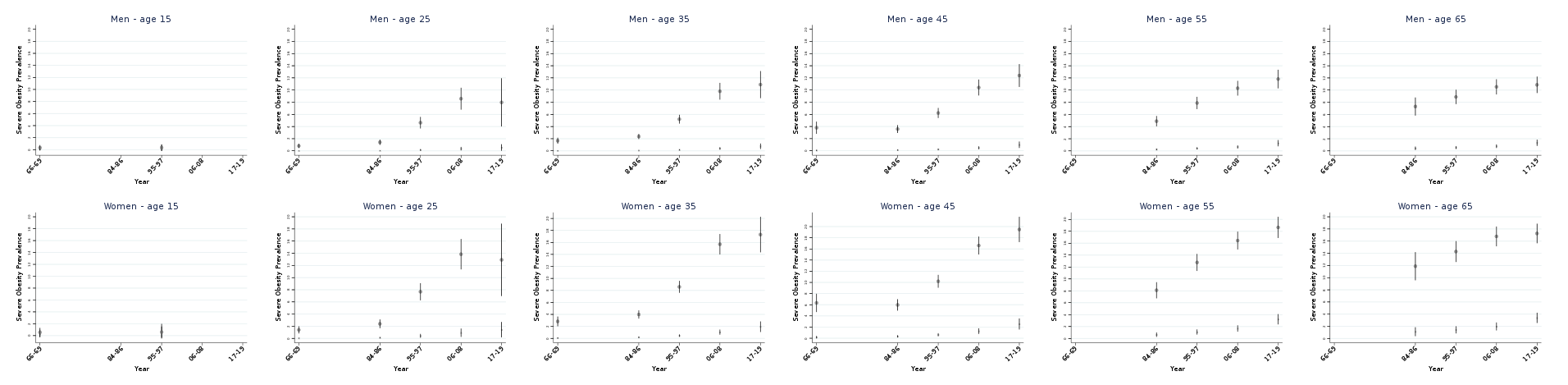


Fig C Estimated prevalence (%, with 95% confidence interval) of severe obesity (BMI ≥ 35 kg/m^2^) by top (most susceptible, circle) and bottom tenth (least susceptible, x) of genome-wide polygenic score by age and time point for 31,717 men and 35,393 women who participated in the HUNT Study, Norway *Youngest observed age in 2017-19 was 28.6 years.


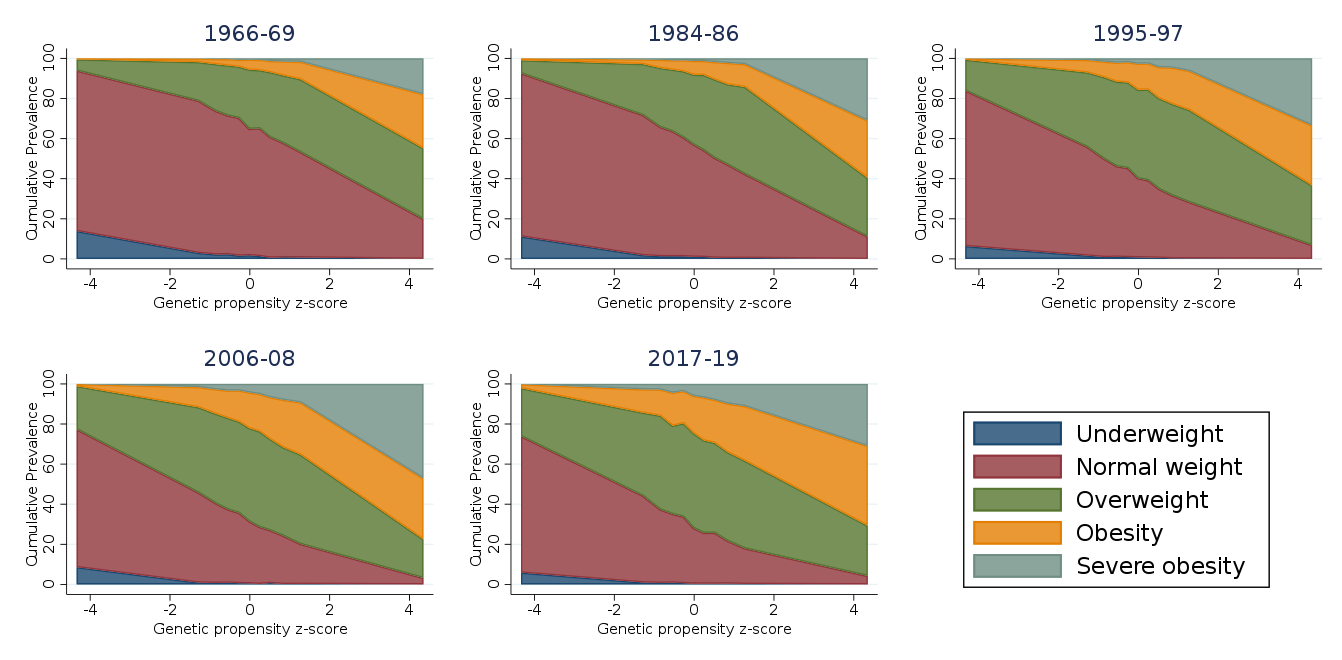
 Fig D Cumulative prevalence of underweight, normal weight, overweight, obesity and severe obesity for the entire study population by time point.


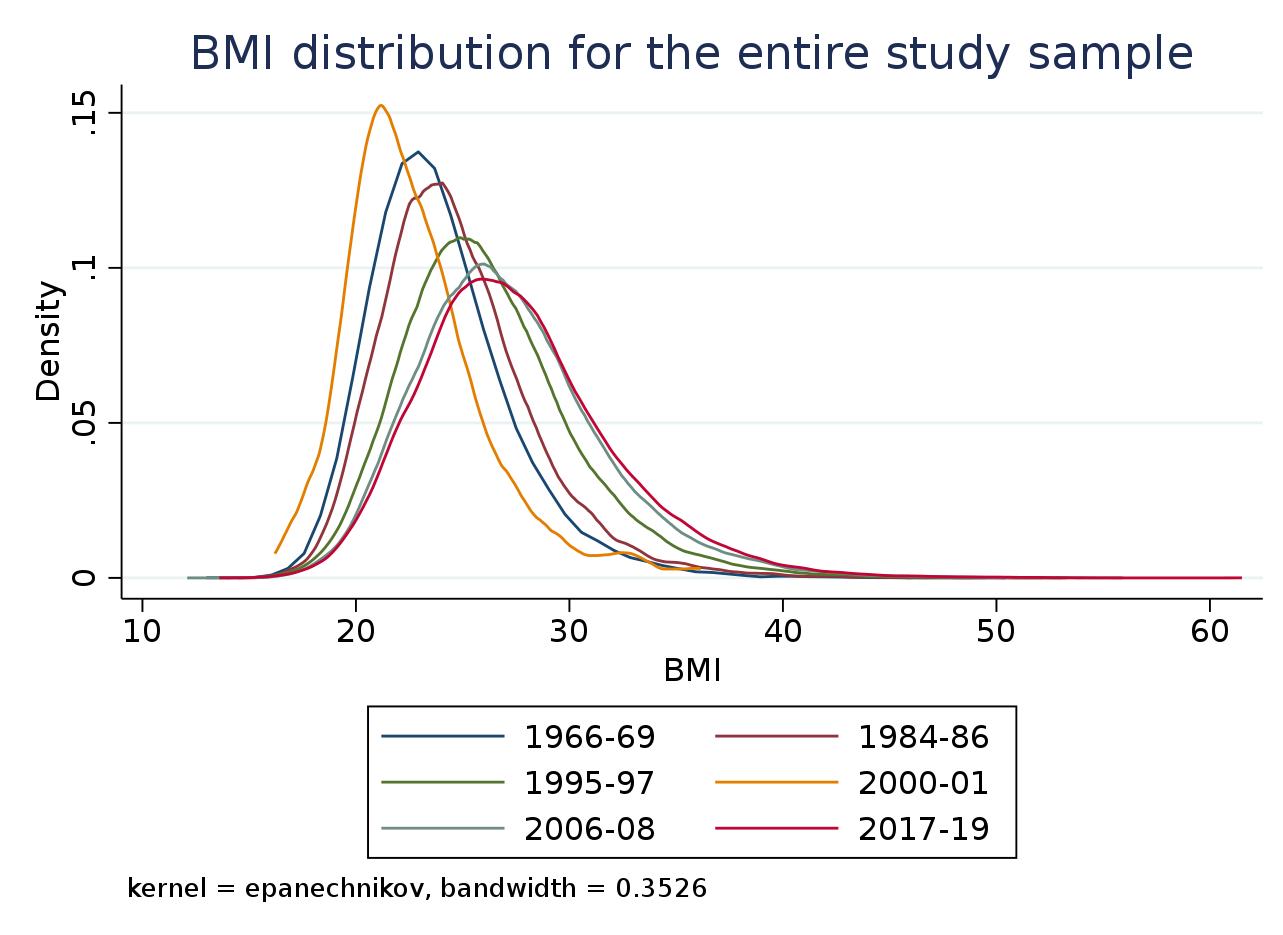


Fig E Kernel plot of BMI distribution for the entire study sample by time of measurement. Participants were age 14-53 in 1966-69, age 20-70 in 1984-86, age 13-79 in 1995-97, age 16-20 in 2000-01, age 18-79 in 2006-08, and age 28-79 in 2017-19.


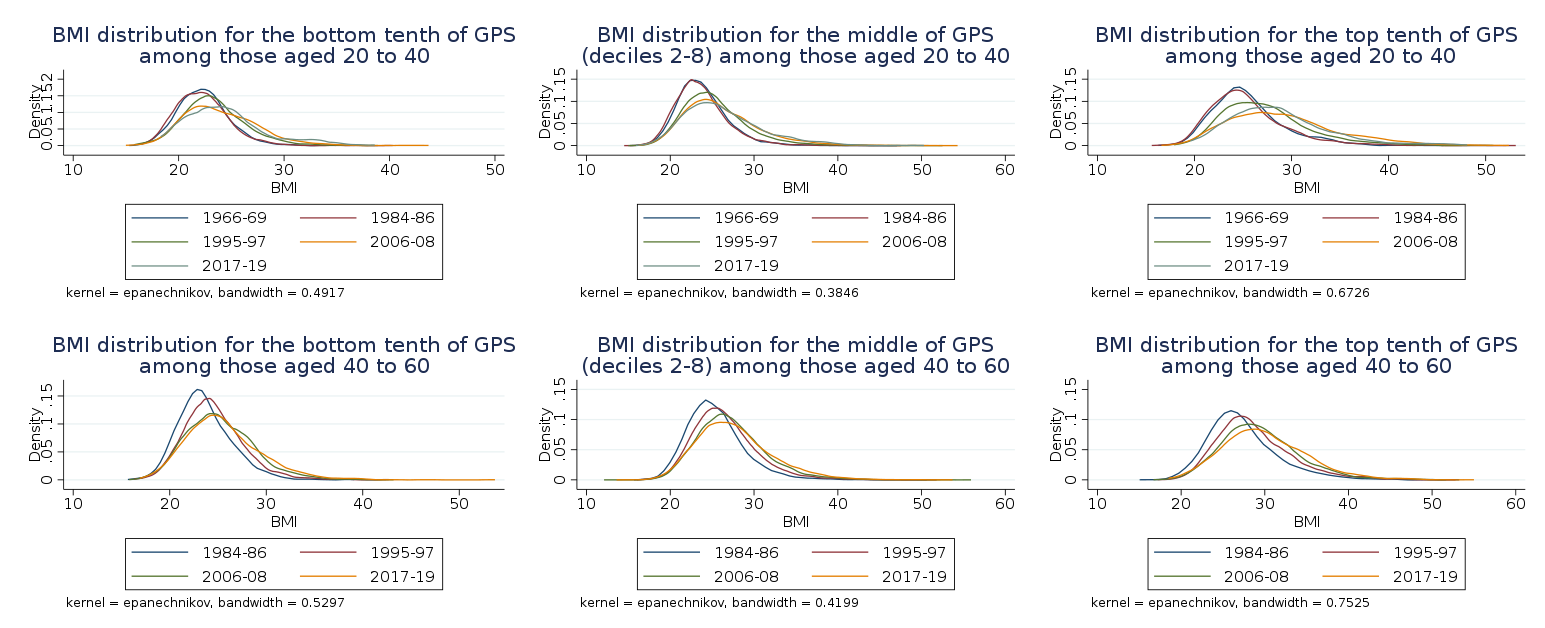


Fig F Kernel plot of BMI distribution by time of measurement, age and categories of genome-wide polygenic score distribution (GPS).


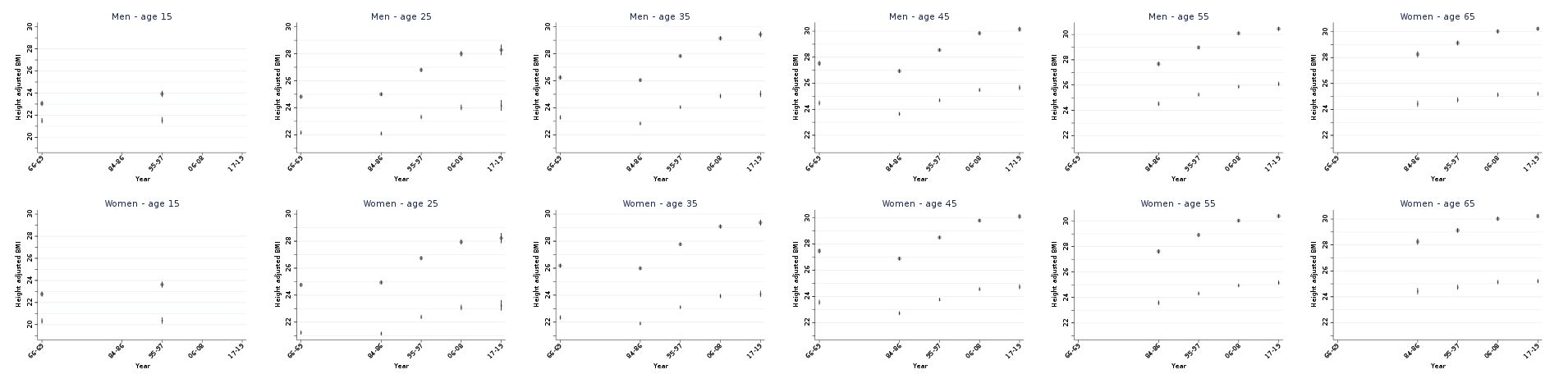


Fig G Estimated height-adjusted BMI (with 95% confidence interval) by top (most susceptible, circle) and bottom tenth (least susceptible, x) of genome-wide polygenic score by age and time point for 31,717 men and 35,393 women who participated in the HUNT Study, Norway. *Youngest observed age in 2017-19 was 28.6 years.


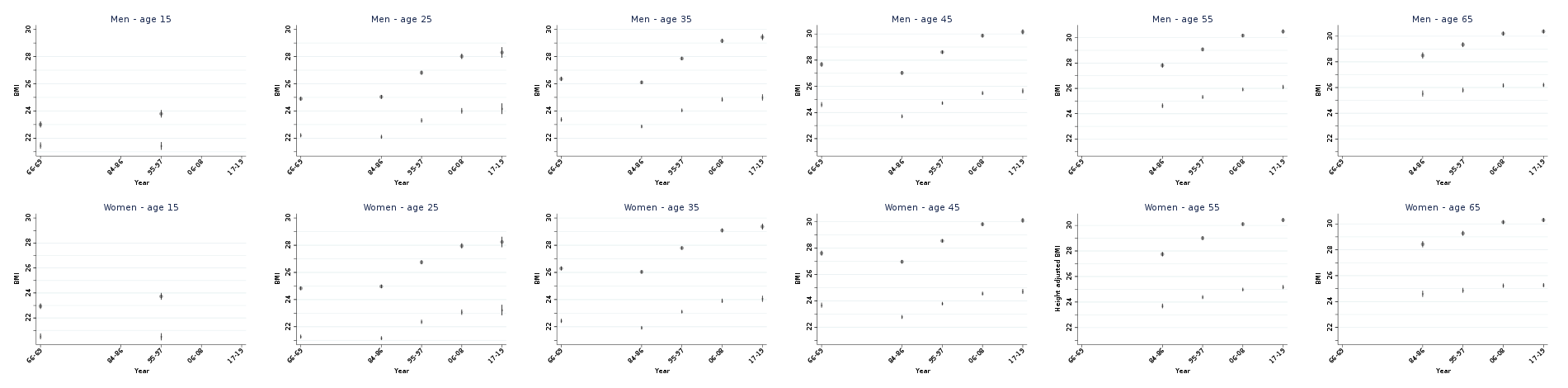


Fig H Estimated BMI (with 95% confidence interval) by top (most susceptible, circle) and bottom tenth (least susceptible, x) of genome-wide polygenic score by age and time point for 31,717 men and 35,393 women who participated in the HUNT Study, Norway. *Youngest observed age in 2017-19 was 28.6 years.


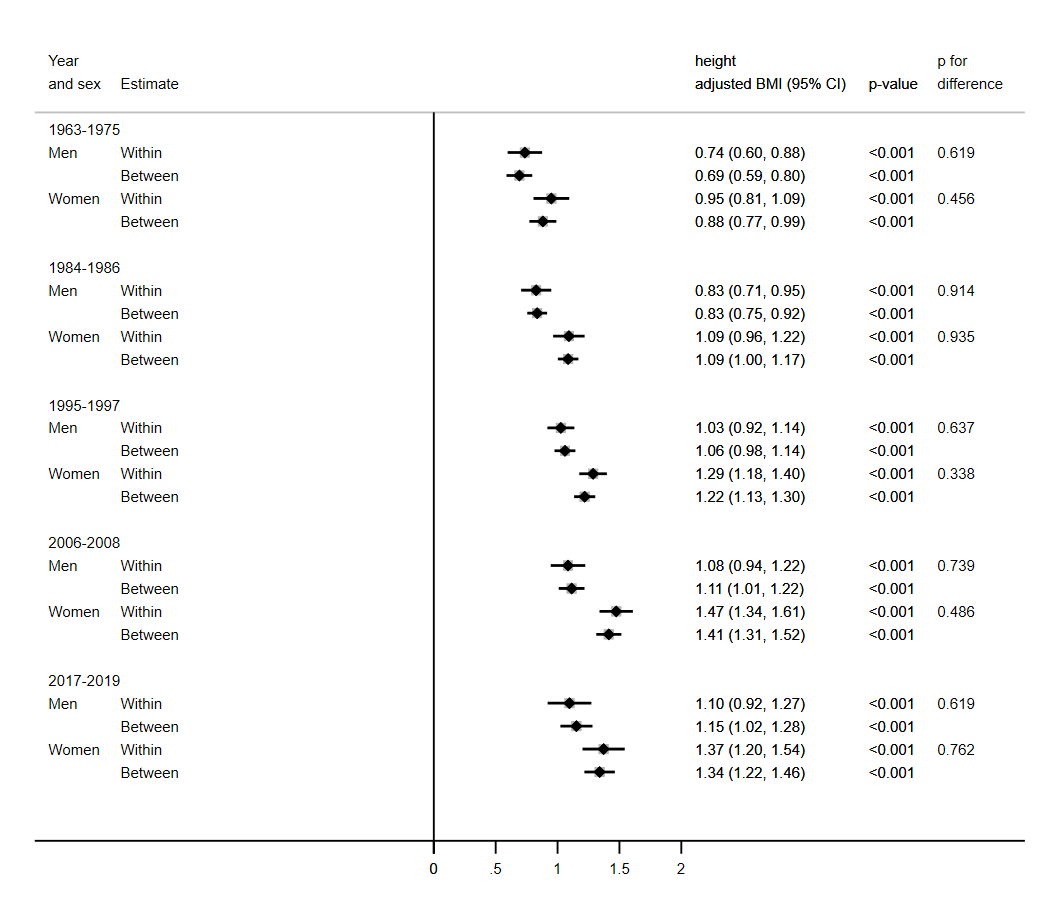
Fig I Estimated association between one standard unit higher GPS for height-adjusted BMI (with 95% confidence interval) within and between siblings by year. Based on 29,585 individuals composing 11,857 sibling groups within participants in the HUNT Study.


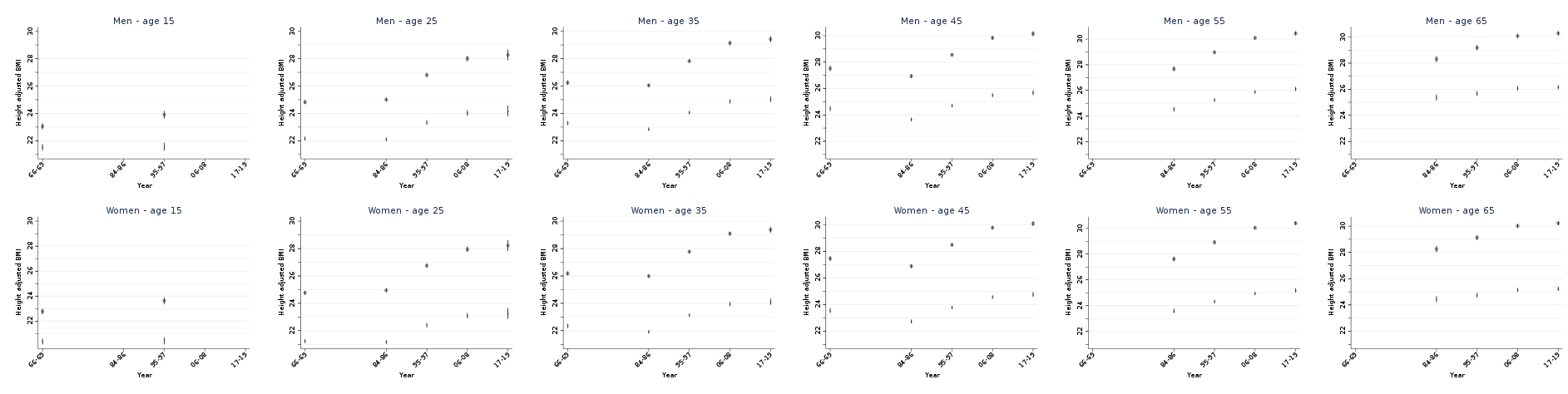


Fig J Estimated BMI (with 95% confidence interval) using data from first degree relatives by top (most susceptible, circle) and bottom tenth (least susceptible, x) of genome-wide polygenic score by age and time point for 31,717 men and 35,393 women who participated in the HUNT Study, Norway. *Youngest observed age in 2017-19 was 28.6 years.


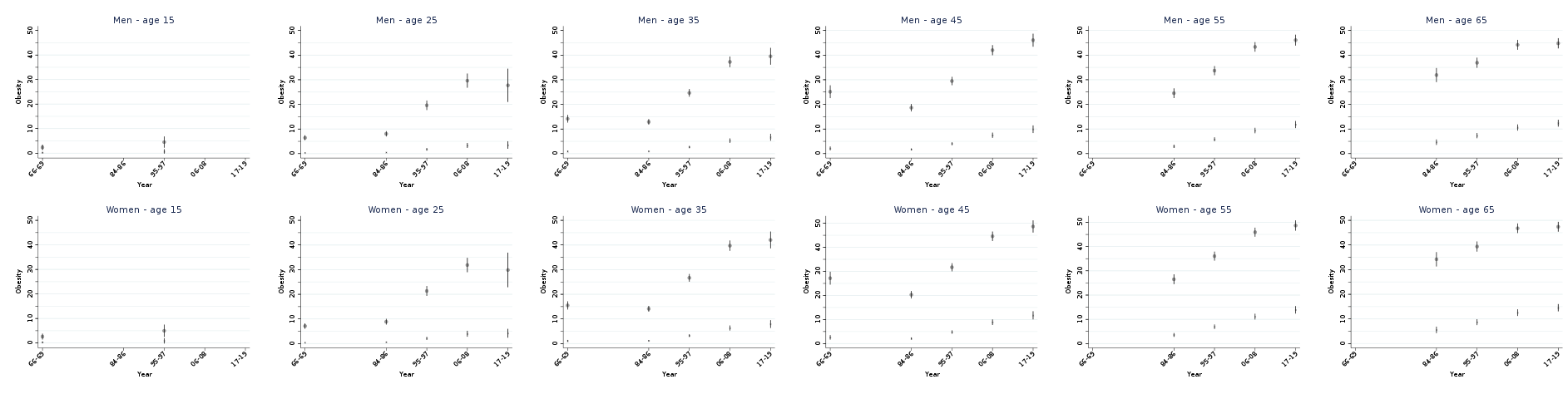


Fig K Estimated prevalence (%, with 95% confidence interval) of obesity (BMI ≥ 30 kg/m^2^) using data from first degree relatives by top (most susceptible, circle) and bottom tenth (least susceptible, x) of genome-wide polygenic score by age and time point for 31,717 men and 35,393 women who participated in the HUNT Study, Norway. *Youngest observed age in 2017-19 was 28.6 years.


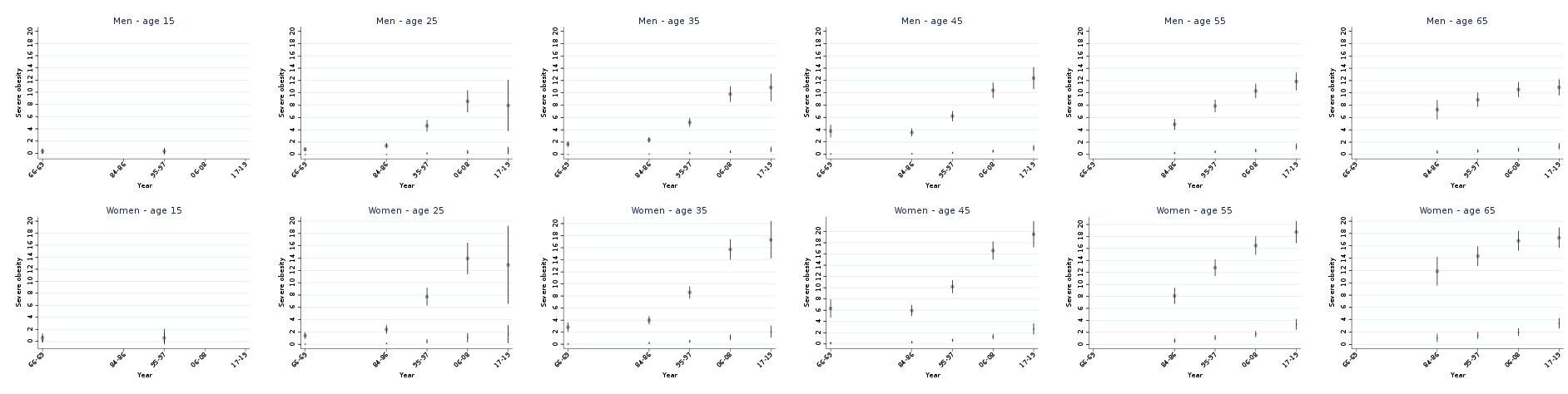


Fig L Estimated prevalence (%, with 95% confidence interval) of severe obesity (BMI ≥ 35 kg/m^2^) using data from first degree relatives by top (most susceptible, circle) and bottom tenth (least susceptible, x) of genome-wide polygenic score by age and time point for 31,717 men and 35,393 women who participated in the HUNT Study, Norway. *Youngest observed age in 2017-19 was 28.6 years.

**Table A The distribution of participants according to both the** **genome-wide polygenic score (GPS) and genetic risk score (GRS).**

|  | GPS decile 0 | GPS decile 1 | GPS decile 2 | GPS decile 3 | GPS decile 4 | GPS decile 5 | GPS decile 6 | GPS decile 7 | GPS decile 8 | GPS decile 9 | GPS Total |
| --- | --- | --- | --- | --- | --- | --- | --- | --- | --- | --- | --- |
| GRS decile 0 | 2,245 | 1,324 | 957 | 737 | 557 | 408 | 298 | 214 | 134 | 49 | 6,923 |
| GRS decile 1 | 1,313 | 1,167 | 1,014 | 838 | 714 | 615 | 485 | 406 | 240 | 120 | 6,912 |
| GRS decile 2 | 958 | 1,054 | 934 | 836 | 786 | 674 | 596 | 508 | 364 | 212 | 6,922 |
| GRS decile 3 | 715 | 843 | 859 | 864 | 846 | 771 | 663 | 569 | 504 | 286 | 6,920 |
| GRS decile 4 | 579 | 720 | 788 | 865 | 783 | 763 | 730 | 675 | 631 | 391 | 6,925 |
| GRS decile 5 | 420 | 612 | 659 | 735 | 790 | 805 | 877 | 763 | 716 | 549 | 6,926 |
| GRS decile 6 | 319 | 457 | 623 | 623 | 750 | 819 | 858 | 907 | 865 | 704 | 6,925 |
| GRS decile 7 | 204 | 376 | 507 | 617 | 703 | 769 | 884 | 968 | 947 | 948 | 6,923 |
| GRS decile 8 | 118 | 254 | 380 | 503 | 585 | 740 | 835 | 974 | 1,159 | 1,373 | 6,921 |
| GRS decile 9 | 51 | 113 | 201 | 303 | 406 | 557 | 696 | 937 | 1,360 | 2,290 | 6,914 |
| GRS Total | 6,922 | 6,920 | 6,922 | 6,921 | 6,920 | 6,921 | 6,922 | 6,921 | 6,920 | 6,922 | 69211 |

**Table B Mean BMI for the bottom and top tenth of genetic predisposition according to the genome-wide polygenic score (GPS) and the genetic risk score (GRS).**

| 1995-97 | Genetic score | Decile | Observations | Mean BMI | Std. Dev. | Min | Max |
| --- | --- | --- | --- | --- | --- | --- | --- |
|  | GRS | 0 | 5,637 | 25.3 | 3.7 | 14.8 | 46.9 |
|  | GPS | 0 | 5,614 | 24.4 | 3.3 | 15.8 | 44.4 |
|  | GRS | 9 | 5,599 | 27.5 | 4.4 | 16.4 | 53.3 |
|  | GPS | 9 | 5,484 | 28.4 | 4.6 | 15.8 | 53.3 |

**Table C Estimated difference BMI between the tenths with highest and lowest genetic susceptibility at various time points.**

|  |  | Men |  |  |  |  |  |  | Women |  |  |  |  |  |
| --- | --- | --- | --- | --- | --- | --- | --- | --- | --- | --- | --- | --- | --- | --- |
| Time range | Age | Difference in BMI | |  | 95% CI |  | p-value |  | Difference in BMI | |  | 95% CI |  | p-value |
| 1966-69 | 25 | 2.69 |  | 2.48 | to | 2.89 | <0.001 |  | 3.56 |  | 3.36 | to | 3.76 | <0.001 |
|  | 35 | 2.99 |  | 2.77 | to | 3.21 | <0.001 |  | 3.86 |  | 3.64 | to | 4.07 | <0.001 |
|  | 45 | 3.07 |  | 2.81 | to | 3.33 | <0.001 |  | 3.94 |  | 3.68 | to | 4.20 | <0.001 |
| 1984-86 | 25 | 2.94 |  | 2.74 | to | 3.13 | <0.001 |  | 3.81 |  | 3.62 | to | 4.00 | <0.001 |
|  | 35 | 3.24 |  | 3.06 | to | 3.42 | <0.001 |  | 4.11 |  | 3.94 | to | 4.28 | <0.001 |
|  | 45 | 3.32 |  | 3.12 | to | 3.52 | <0.001 |  | 4.19 |  | 3.99 | to | 4.39 | <0.001 |
|  | 55 | 3.18 |  | 2.93 | to | 3.43 | <0.001 |  | 4.05 |  | 3.80 | to | 4.30 | <0.001 |
|  | 65 | 2.97 |  | 2.65 | to | 3.29 | <0.001 |  | 3.84 |  | 3.52 | to | 4.16 | <0.001 |
| 1995-97 | 25 | 3.51 |  | 3.29 | to | 3.73 | <0.001 |  | 4.38 |  | 4.17 | to | 4.59 | <0.001 |
|  | 35 | 3.81 |  | 3.63 | to | 3.99 | <0.001 |  | 4.68 |  | 4.51 | to | 4.86 | <0.001 |
|  | 45 | 3.89 |  | 3.71 | to | 4.07 | <0.001 |  | 4.76 |  | 4.59 | to | 4.94 | <0.001 |
|  | 55 | 3.75 |  | 3.54 | to | 3.96 | <0.001 |  | 4.62 |  | 4.42 | to | 4.83 | <0.001 |
|  | 65 | 3.54 |  | 3.27 | to | 3.80 | <0.001 |  | 4.41 |  | 4.14 | to | 4.67 | <0.001 |
| 2006-08 | 25 | 4.01 |  | 3.74 | to | 4.28 | <0.001 |  | 4.88 |  | 4.62 | to | 5.14 | <0.001 |
|  | 35 | 4.31 |  | 4.08 | to | 4.53 | <0.001 |  | 5.18 |  | 4.96 | to | 5.39 | <0.001 |
|  | 45 | 4.39 |  | 4.19 | to | 4.59 | <0.001 |  | 5.26 |  | 5.07 | to | 5.45 | <0.001 |
|  | 55 | 4.25 |  | 4.05 | to | 4.45 | <0.001 |  | 5.12 |  | 4.93 | to | 5.31 | <0.001 |
|  | 65 | 4.04 |  | 3.81 | to | 4.27 | <0.001 |  | 4.91 |  | 4.68 | to | 5.13 | <0.001 |
| 2017-19 | 25 | 3.51 |  | 3.29 | to | 3.73 | <0.001 |  | 4.38 |  | 4.17 | to | 4.59 | <0.001 |
|  | 35 | 3.81 |  | 3.63 | to | 3.99 | <0.001 |  | 4.68 |  | 4.51 | to | 4.86 | <0.001 |
|  | 45 | 3.89 |  | 3.71 | to | 4.07 | <0.001 |  | 4.76 |  | 4.59 | to | 4.94 | <0.001 |
|  | 55 | 3.75 |  | 3.54 | to | 3.96 | <0.001 |  | 4.62 |  | 4.42 | to | 4.83 | <0.001 |
|  | 65 | 3.54 |  | 3.27 | to | 3.80 | <0.001 |  | 4.41 |  | 4.14 | to | 4.67 | <0.001 |

**Table D Estimated difference in height adjusted BMI over time for the tenths with the highest and lowest genetic susceptibility for men and women combined.** Calculated as the difference between the estimated height adjusted BMI at the earliest time point subtracted from the estimated prevalence at the most recent time point. Genome-wide polygenic score (GPS) and Body mass index (BMI).

| Time range | Age | GPS decile | BMI |  | 95% CI |  | p-value |
| --- | --- | --- | --- | --- | --- | --- | --- |
| 1966-2019 | 25 | 0 | 2.01 | 1.61 | to | 2.41 | <0.001 |
|  |  | 9 | 3.46 | 3.06 | to | 3.86 | <0.001 |
|  | 35 | 0 | 1.74 | 1.46 | to | 2.01 | <0.001 |
|  |  | 9 | 3.18 | 2.91 | to | 3.45 | <0.001 |
|  | 45 | 0 | 1.18 | 0.92 | to | 1.45 | <0.001 |
|  |  | 9 | 2.63 | 2.37 | to | 2.89 | <0.001 |
| 1984-2019 | 55 | 0 | 1.57 | 1.37 | to | 1.78 | <0.001 |
|  |  | 9 | 2.77 | 2.57 | to | 2.97 | <0.001 |
|  | 65 | 0 | 0.77 | 0.55 | to | 0.99 | <0.001 |
|  |  | 9 | 1.97 | 1.75 | to | 2.19 | <0.001 |

**Table E Estimated difference in BMI over time for the tenths with the highest and lowest genetic susceptibility for men and women combined.** Calculated as the difference between the estimated height adjusted BMI at the earliest time point subtracted from the estimated prevalence at the most recent time point. Genome-wide polygenic score (GPS) and Body mass index (BMI).

| Time range | Age | GPS decile | BMI |  | 95% CI |  | p-value |
| --- | --- | --- | --- | --- | --- | --- | --- |
| 1966-2019 | 25 | 0 | 1.09 | 0.93 | to | 1.25 | <0.001 |
|  |  | 9 | 1.91 | 1.75 | to | 2.07 | <0.001 |
|  | 35 | 0 | 0.68 | 0.52 | to | 0.83 | <0.001 |
|  |  | 9 | 1.50 | 1.34 | to | 1.65 | <0.001 |
|  | 45 | 0 | 0.12 | -0.05 | to | 0.28 | 0.160 |
|  |  | 9 | 0.94 | 0.78 | to | 1.10 | <0.001 |
| 1984-2019 | 55 | 0 | 0.69 | 0.59 | to | 0.79 | <0.001 |
|  |  | 9 | 1.26 | 1.16 | to | 1.36 | <0.001 |
|  | 65 | 0 | 0.27 | 0.14 | to | 0.39 | <0.001 |
|  |  | 9 | 0.84 | 0.71 | to | 0.96 | <0.001 |

**Table F Estimated difference in BMI between the tenths with the highest and lowest genetic susceptibility over time for all ages.**

| Time range | BMI difference | 95% CI | | | p-value |
| --- | --- | --- | --- | --- | --- |
| 1966-1986 | 0.25 | 0.09 | to | 0.41 | 0.002 |
| 1966-1997 | 0.82 | 0.61 | to | 1.03 | <0.001 |
| 1966-2008 | 1.32 | 1.04 | to | 1.60 | <0.001 |
| 1966-2019 | 1.45 | 1.09 | to | 1.81 | <0.001 |

**Table G Estimates from sibling analysis with BMI. Body mass index (BMI). Difference is the between sibship coefficient minus the within sibship coefficient.**

|  |  | Men |  |  |  |  | Women |  |  |  |  |
| --- | --- | --- | --- | --- | --- | --- | --- | --- | --- | --- | --- |
| Year |  | BMI |  | 95% CI |  | p-value | BMI |  | 95% CI |  | p-value |
| 1966-69 | Between sibships | 0.69 | 0.59 | to | 0.80 | <0.001 | 0.88 | 0.77 | to | 0.99 | <0.001 |
|  | Within sibships | 0.74 | 0.60 | to | 0.88 | <0.001 | 0.95 | 0.81 | to | 1.09 | <0.001 |
|  | Difference | -0.04 | -0.22 | to | 0.13 | 0.619 | -0.07 | -0.25 | to | 0.11 | 0.456 |
| 1984-86 | Between sibships | 0.83 | 0.75 | to | 0.92 | <0.001 | 1.09 | 1.00 | to | 1.17 | <0.001 |
|  | Within sibships | 0.83 | 0.71 | to | 0.95 | <0.001 | 1.09 | 0.96 | to | 1.22 | <0.001 |
|  | Difference | 0.01 | -0.14 | to | 0.15 | 0.914 | -0.01 | -0.16 | to | 0.15 | 0.935 |
| 1995-97 | Between sibships | 1.06 | 0.98 | to | 1.14 | <0.001 | 1.22 | 1.13 | to | 1.30 | <0.001 |
|  | Within sibships | 1.03 | 0.92 | to | 1.14 | <0.001 | 1.29 | 1.18 | to | 1.40 | <0.001 |
|  | Difference | 0.03 | -0.11 | to | 0.17 | 0.637 | -0.07 | -0.21 | to | 0.07 | 0.338 |
| 2006-08 | Between sibships | 1.11 | 1.01 | to | 1.22 | <0.001 | 1.41 | 1.31 | to | 1.52 | <0.001 |
|  | Within sibships | 1.08 | 0.94 | to | 1.22 | <0.001 | 1.47 | 1.34 | to | 1.61 | <0.001 |
|  | Difference | 0.03 | -0.14 | to | 0.20 | 0.739 | -0.06 | -0.23 | to | 0.11 | 0.486 |
| 2017-19 | Between sibships | 1.15 | 1.02 | to | 1.28 | <0.001 | 1.34 | 1.22 | to | 1.46 | <0.001 |
|  | Within sibships | 1.10 | 0.92 | to | 1.27 | <0.001 | 1.37 | 1.20 | to | 1.54 | <0.001 |
|  | Difference | 0.06 | -0.16 | to | 0.28 | 0.619 | -0.03 | -0.24 | to | 0.18 | 0.762 |

**Table H Estimates from sibling analysis with obesity. Odds ratio (OR). Difference is the between sibship coefficient minus the within sibship coefficient.**

|  |  | Men |  |  |  |  | Women |  |  |  |  |
| --- | --- | --- | --- | --- | --- | --- | --- | --- | --- | --- | --- |
| Year |  | OR |  | 95% CI |  | p-value | OR |  | 95% CI |  | p-value |
| 1966-69 | Between sibships | 2.46 | 1.79 | to | 3.37 | <0.001 | 2.28 | 1.81 | to | 2.86 | <0.001 |
|  | Within sibships | 3.18 | 1.99 | to | 5.09 | <0.001 | 2.12 | 1.52 | to | 2.95 | <0.001 |
|  | Difference | 0.77 | 0.45 | to | 1.33 | 0.354 | 1.08 | 0.73 | to | 1.58 | 0.711 |
| 1984-86 | Between sibships | 2.21 | 1.92 | to | 2.54 | <0.001 | 2.13 | 1.87 | to | 2.43 | <0.001 |
|  | Within sibships | 2.02 | 1.66 | to | 2.46 | <0.001 | 2.09 | 1.74 | to | 2.51 | <0.001 |
|  | Difference | 1.09 | 0.86 | to | 1.38 | 0.464 | 1.02 | 0.82 | to | 1.26 | 0.869 |
| 1995-97 | Between sibships | 2.15 | 1.97 | to | 2.34 | <0.001 | 2.05 | 1.89 | to | 2.22 | <0.001 |
|  | Within sibships | 2.15 | 1.91 | to | 2.41 | <0.001 | 1.87 | 1.68 | to | 2.09 | <0.001 |
|  | Difference | 1.00 | 0.87 | to | 1.15 | 0.993 | 1.09 | 0.96 | to | 1.25 | 0.187 |
| 2006-08 | Between sibships | 2.12 | 1.96 | to | 2.30 | <0.001 | 2.03 | 1.88 | to | 2.19 | <0.001 |
|  | Within sibships | 2.00 | 1.79 | to | 2.23 | <0.001 | 2.00 | 1.80 | to | 2.21 | <0.001 |
|  | Difference | 1.06 | 0.93 | to | 1.21 | 0.36 | 1.02 | 0.90 | to | 1.15 | 0.810 |
| 2017-19 | Between sibships | 2.00 | 1.83 | to | 2.19 | <0.001 | 1.89 | 1.74 | to | 2.05 | <0.001 |
|  | Within sibships | 1.90 | 1.68 | to | 2.14 | <0.001 | 1.72 | 1.53 | to | 1.93 | <0.001 |
|  | Difference | 1.05 | 0.91 | to | 1.22 | 0.478 | 1.10 | 0.96 | to | 1.26 | 0.174 |

**Table I Estimated difference in natural logarithm of BMI (lnBMI) between the tenths with the highest and lowest genetic susceptibility over time.** The estimated differences in lnBMI as well as the confidence interval limits were exponentiated to illustrate the estimated difference from a logarithmic model expressed in BMI units.

| Time range | lnBMI | 95% CI |  |  | p-value | BMI | 95% CI |  |
| --- | --- | --- | --- | --- | --- | --- | --- | --- |
| 1966-1986 | 0.01 | 0.00 to | 0.02 |  | 0.001 | 1.01 | 1.00 to | 1.02 |
| 1966-1997 | 0.03 | 0.02 to | 0.03 |  | <0.001 | 1.03 | 1.02 to | 1.03 |
| 1966-2008 | 0.04 | 0.03 to | 0.05 |  | <0.001 | 1.04 | 1.03 to | 1.05 |
| 1966-2019 | 0.04 | 0.03 to | 0.06 |  | <0.001 | 1.04 | 1.03 to | 1.06 |
