# Supplementary material for: Genetic associations with temporal shifts in obesity and severe obesity during the obesity epidemic in Norway: A longitudinal population-based cohort (the HUNT Study)
Source: PLoS Med. 2020 Dec 14;17(12):e1003452. doi: 10.1371/journal.pmed.1003452 (PMC7735641; doi:10.1371/journal.pmed.1003452)
Supplement: S1 Checklist — (DOC) [file pmed.1003452.s004.doc]

STROBE Statement—checklist of items that should be included in reports of observational studies

|  | Item No | Recommendation |
| --- | --- | --- |
| **Title and abstract** | 1 | (*a*) Indicate the study’s design with a commonly used term in the title or the abstract  Abstract: (Methods and Findings), paragraph 2  *67,110 individuals aged 13-80 years in the Nord-Trøndelag Region of Norway participated with repeated standardized BMI measurements from 1966 to 2019 and were genotyped in a longitudinal population based health study (The Trøndelag Health Study, HUNT).* |
|  |  | (*b*) Provide in the abstract an informative and balanced summary of what was done and what was found  Abstract: (Methods and Findings), paragraph 2  *Linear mixed models with observations nested within individuals were used to model the association between a genome-wide polygenic score for BMI and BMI while generalized estimating equations were used for obesity (BMI ≥ 30 kg/m^2^) and severe obesity (BMI ≥ 35 kg/m^2^).* |
| Introduction | | |
| Background/rationale | 2 | Explain the scientific background and rationale for the investigation being reported  Introduction, paragraph 3  *Hence, the goal of this study is to quantify and validate the interplay between our genes and the environment.* |
| Objectives | 3 | State specific objectives, including any prespecified hypotheses  Introduction, paragraph 2  *Utilizing the dramatic changes in our environment from 1966 to 2019, we now apply the more powerful GPS to show that the same trends exist with the prevalence of obesity.*  Introduction, paragraph 3  *As the GPS distribution between siblings sharing a mother and father is random, we use sibling design to compare BMI within families.* |
| Methods | | |
| Study design | 4 | Present key elements of study design early in the paper  Methods, paragraph 1  *The study population consists of participants from the HUNT Study (1984-2019) linked to previous height and weight measurements in the tuberculosis screening program (1966-69). Please see abstract paragraph 2.* |
| Setting | 5 | Describe the setting, locations, and relevant dates, including periods of recruitment, exposure, follow-up, and data collection  Methods, paragraph 1  *The entire adult population in the Nord-Trøndelag Region was invited to participate in the HUNT Study conducted in four waves: HUNT1 (1984-86), HUNT2 (1995-97), HUNT3 (2006-08) and HUNT4 (2017-19). The Young-HUNT Study, recruiting all teenagers aged 13-19 in the Nord-Trøndelag Region, was conducted in 1995-97, 2000-01, 2006-08 and 2017-19.*  Methods, paragraph 1  *The tuberculosis screening program was established in 1943 and contributed to the surveillance of tuberculosis in the general Norwegian population.* *[1] From the tuberculosis screening data, we limited height and weight data to the time interval with most observations from 1966-69 and excluded participants younger than 14 years as they were not targets for total population surveillance.* |
| Participants | 6 | (*a*) *Cohort study*—Give the eligibility criteria, and the sources and methods of selection of participants. Describe methods of follow-up  Methods, paragraph 1  *The entire adult population in the Nord-Trøndelag Region was invited to participate in the HUNT Study*  Methods, paragraph 1  *The Young-HUNT Study, recruiting all teenagers aged 13-19 in the Nord-Trøndelag Region*  Methods, paragraph  *The tuberculosis screening program was established in 1943 and contributed to the surveillance of tuberculosis in the general Norwegian population [1]. We limited height and weight data to the time interval with most observations from 1966-69 and excluded participants younger than 14 years as they were not targets for total population surveillance.*  Methods (Genotyping and Computation of Genome-wide Polygenice Score (GPS), paragraph 3  *Genetic analyses were performed on blood samples collected from adults participating in HUNT2 and HUNT3 [2].*  *Case-control study*—Give the eligibility criteria, and the sources and methods of case ascertainment and control selection. Give the rationale for the choice of cases and controls  *Cross-sectional study*—Give the eligibility criteria, and the sources and methods of selection of participants |
|  |  | (*b*) *Cohort study*—For matched studies, give matching criteria and number of exposed and unexposed  N/A  *Case-control study*—For matched studies, give matching criteria and the number of controls per case |
| Variables | 7 | Clearly define all outcomes, exposures, predictors, potential confounders, and effect modifiers. Give diagnostic criteria, if applicable  Methods, (Statistical analyses), paragraph 5  *We then estimated the effect of genetic risk of obesity on height-adjusted BMI according to time of measurement, age and sex.*  Methods, (Statistical analyses), paragraph 6  *We modelled the association of GPS with obesity and severe obesity using generalized estimating equations.*  Methods, (Statistical analyses), paragraph 7  *To assess whether assortative mating, dynastic effects or population stratification influenced the results, we analysed the association of the GPS with height-adjusted BMI as well as with the prevalence of obesity within and between siblings.* |
| Data sources/ measurement | 8* | For each variable of interest, give sources of data and details of methods of assessment (measurement). Describe comparability of assessment methods if there is more than one group  Methods, (BMI Assessment), paragraph 2  *Measurements were standardized with weight measured to the nearest half kilogram with the participants wearing light clothes and no shoes and height measured to the nearest centimetre [3]. BMI was calculated using the formula weight in kilograms per metre squared. As defined by the World Health Organization, we refer to overweight as BMI greater than or equal to 25 and to obesity as BMI greater than or equal to 30 [4]. We chose to refer to severe obesity as BMI greater than or equal to 35. As previously described, we calculated BMI z-scores for participants younger than 18 years [5].* *In the statistical analyses, we adjusted BMI for height to account for any effect of the six centimetre height increase in the population since the 1960s [6].* *As suggested by the reviewers, we repeated the analyses using BMI without adjustment for height and compared the results.*  Methods, (Genotyping and Computation of Genome-wide Polygenice Score (GPS), paragraph 3  *We constructed a GPS using weights from the polygenic score for BMI derived and validated by Khera et al.*  Methods, (Genotyping and Computation of Genome-wide Polygenice Score (GPS), paragraph 3  *We included 2.07 million of the 2.1 million common variants, excluding those with insufficient quality of genotyping or imputation in HUNT (r^2^<0.8).* |
| Bias | 9 | Describe any efforts to address potential sources of bias  Methods, (Statistical Analyses), paragraph 7  *To assess whether assortative mating, dynastic effects or population stratification influenced the results, we analysed the association of the GPS with height-adjusted BMI as well as with the prevalence of obesity within and between siblings.*  Methods, (Statistical Analyses), paragraph 8  *To assess the possibility of selection bias, we estimated the association between obesity status in the 1960s and availability of genetic data*  Methods, (Statistical Analyses), paragraph 8  *We used genetic data from first degree relatives to evaluate if exclusions due to missing genetic data biased the results.* |
| Study size | 10 | Explain how the study size was arrived at  Methods, paragraph 1  *Our study includes 67,110 individuals of European descent aged 13-80.*  *Please see Fig 1* |
| Quantitative variables | 11 | Explain how quantitative variables were handled in the analyses. If applicable, describe which groupings were chosen and why  Methods, (Statistical Analyses), paragraph 5  *To assess linearity, we modelled the association between the GPS and BMI using linear splines with nine knots according to percentiles of the distribution. We adjusted for sex and time of measurement as categorical variables and used linear splines with knots at every 20 years to adjust for age. We also adjusted for 20 principal components and genotyping batch. Further, we allowed the effect of the GPS to differ according to time of measurement, sex, and age using interaction terms for each. Although we adjusted for age using splines, we used 20-year age categories for the interaction terms. The association between the GPS and BMI was fairly linear justifying a linearity assumption for GPS (S1 Fig). Hence, for the main analyses, we divided the study population into ten equally sized groups, the top tenth being the most genetically susceptible to higher BMI and the bottom tenth being the least genetically susceptible. We then estimated the effect of genetic risk of obesity on height-adjusted BMI according to time of measurement, age and sex. In addition to the previously described interaction terms, we included an interaction term between age and time of measurement.*  Methods, (Statistical Analyses), paragraph 6  *We modelled the association of GPS with obesity and severe obesity using generalized estimating equations. We included the same covariates as in the models assessing height-adjusted BMI.* |
| Statistical methods | 12 | (*a*) Describe all statistical methods, including those used to control for confounding  Please see text answer for question 11 and question 9 |
|  |  | (*b*) Describe any methods used to examine subgroups and interactions |
|  |  | (*c*) Explain how missing data were addressed |
|  |  | (*d*) *Cohort study*—If applicable, explain how loss to follow-up was addressed  *Case-control study*—If applicable, explain how matching of cases and controls was addressed  *Cross-sectional study*—If applicable, describe analytical methods taking account of sampling strategy |
|  |  | (*e*) Describe any sensitivity analyses  Methods, (Statistical Analyses), paragraph 7  *To assess whether assortative mating, dynastic effects or population stratification influenced the results, we analysed the association of the GPS with height-adjusted BMI as well as with the prevalence of obesity within and between siblings. The sibships’ GPS average and each sibling’s deviation from the group GPS average were calculated and included as independent variables in the regression, where the within sibship coefficient is an estimate for differentially genetically exposed siblings. Between sibship coefficients exceeding the within sibship coefficients would indicate confounding at the sibship level. Unlike the main analyses, we performed these models separately by time point with one observation per individual, assuming the association of GPS with BMI and with obesity to be linear and constant over different ages.*  Methods, (Statistical Analyses), paragraph 8  *To assess the possibility of selection bias, we estimated the association between obesity status in the 1960s and availability of genetic data. We compared the estimated BMI and prevalence of obesity among 38,378 individuals excluded due to lack of genetic data to the estimated BMI and prevalence of obesity for individuals in our study sample. To approximate the relative rather than the absolute difference in BMI, we assessed the association between the GPS and the natural logarithm of BMI.*  *We used genetic data from first degree relatives to evaluate if exclusions due to missing genetic data biased the results.* *To approximate the relative rather than the absolute difference in BMI, we assessed the association between the GPS and the natural logarithm of BMI.* |

Continued on next page

| Results | | |
| --- | --- | --- |
| Participants | 13* | (a) Report numbers of individuals at each stage of study—eg numbers potentially eligible, examined for eligibility, confirmed eligible, included in the study, completing follow-up, and analysed  Results, paragraph 1 (See Fig 1)  *The study sample consists of 67,110 participants aged 13-80 years with a total of 202 030 BMI measurements, with an average of three measurements per person (Fig 1).* |
|  |  | (b) Give reasons for non-participation at each stage  See Fig 1 |
|  |  | (c) Consider use of a flow diagram  See Fig 1 |
| Descriptive data | 14* | (a) Give characteristics of study participants (eg demographic, clinical, social) and information on exposures and potential confounders  Results, paragraph 1  *The average age of participants increased gradually from 30 years in the 1960s to 60 years in 2017-19, except for 2000-01 when only adolescents participated.* |
|  |  | (b) Indicate number of participants with missing data for each variable of interest |
|  |  | (c) *Cohort study*—Summarise follow-up time (eg, average and total amount)  Results, paragraph 1  *The average age of participants increased gradually from 30 years in the 1960s to 60 years in 2017-19, except for 2000-01 when only adolescents participated* |
| Outcome data | 15* | *Cohort study*—Report numbers of outcome events or summary measures over time  See Fig 1 |
|  |  | *Case-control study—*Report numbers in each exposure category, or summary measures of exposure |
|  |  | *Cross-sectional study—*Report numbers of outcome events or summary measures |
| Main results | 16 | (*a*) Give unadjusted estimates and, if applicable, confounder-adjusted estimates and their precision (eg, 95% confidence interval). Make clear which confounders were adjusted for and why they were included  Results, paragraph 2  *At different ages and decades, estimated height-adjusted BMI differed by 2.5 to 5 BMI units across polygenic score tenths, and the difference varied proportional to the changes in population weight (Table 4, S5 Fig).*  Results, paragraph 3  *Among 35 year old men, the prevalence of obesity for the least predisposed tenth increased from 0.9% (95% confidence interval [CI] 0.6 to 1.2%) to 6.5% (95% CI 5.0 to 8.0%) while for the most predisposed tenth it increased from 14.2% (95% CI 12.6 to 15.7%) to 39.6% (95% CI 36.1 to 43.0%). Hence, the absolute change in prevalence of obesity was 19.8 percentage points (95% CI 16.2 to 23.5, p<0.0001* *percentage points) greater for the highly predisposed. Equivalently for women of the same age, the prevalence of obesity for the least predisposed tenth increased from 1.1% (95% CI 0.7 to1.5%) to 7.6% (95% CI 6.0 to 9.2%) while the most predisposed tenth increased from 15.4% (95% CI 13.7 to 17.2%) to 42.0% (95% CI 38.7 to 45.4%). The absolute change in prevalence of obesity among women was 20.0 percentage points (95% CI 16.4 to 23.7, p<0.0001* *percentage points) greater for the highly predisposed (Fig 3, Table 6, Table 7). A similar trend is evident for severe obesity (Fig 4, Table 6, Table 7); the corresponding absolute change in prevalence of severe obesity for men and women respectively, was 8.5 percentage points (95% CI 6.3 to 10.7, p<0.0001* *percentage points) and 12.6 percentage points (95% CI 9.6 to 15.6, p<0.0001* *percentage points) greater for the highly predisposed.* *Similar yet slightly smaller changes were found among other ages. (Fig 3, Fig 4, Table 6, Table 7).* |
|  |  | (*b*) Report category boundaries when continuous variables were categorized |
|  |  | (*c*) If relevant, consider translating estimates of relative risk into absolute risk for a meaningful time period  Results, paragraph 2  *Using the natural logarithm of BMI as the outcome, we still found larger effect sizes in more recent years (Table I in S2 Text).* |
| Other analyses | 17 | Report other analyses done—eg analyses of subgroups and interactions, and sensitivity analyses  Results, paragraph 2  *We found comparable associations between polygenic risk score and BMI as well as obesity within and between sibling groups with little evidence of bias from assortative mating, population stratification or dynastic effects (Fig 2, Fig G in S2 Text, Table G in S2 Text, Table H in S2 Text). HUNT participants excluded due to missing genetic data had only a slightly higher BMI compared to participants with genetic data [5].Using genetic data from first degree relatives, we found no evidence that exclusion due to missing genetic data biased results (Fig H in S2 Text, Fig I in S2 Text, Fig J in S2 Text).* |
| Discussion | | |
| Key results | 18 | Summarise key results with reference to study objectives  Discussion, paragraph 1  *In this study, we observed that, from the 1960s to the late-2010s, the prevalence of obesity and severe obesity increased dramatically for the genetically predisposed yet remained relatively unchanged for the least genetically predisposed. For 35 year old men and women, the absolute increase in prevalence of obesity using the GPS was 20 percentage points greater for the most genetically predisposed tenth compared with the least predisposed tenth.* *This suggests an increasing genetic inequality in obesity and severe obesity over time, consistent with the increasingly obesogenic environment [7] and the increasing variance in BMI seen over time in many countries [8]. Interestingly, the increase in severe obesity across GPS tenths was strongest among women.* *Conceptualizing year of assessment as a broad indicator of the obesogenic environment, our study illustrates that despite being a very heritable trait, body weight seems modifiable by obesogenic exposure. Further, our findings demonstrate an interplay between genes and the environment that is robust to family-level confounding using sibling design.* |
| Limitations | 19 | Discuss limitations of the study, taking into account sources of potential bias or imprecision. Discuss both direction and magnitude of any potential bias  Discussion, (Strength and limitations of this study), paragraph 4 and 5  *Our study sample is considered unselected and is little affected by non-participation bias or bias from selective survival to date of genetic testing, as previously described [5]. For the eldest cohorts, we acknowledge a weak association between BMI measured in the 1960s and survival to and participation in genetic analyses in the 1990s. Still, we found little evidence of selection bias in analyses using genetic data from first degree relatives as a proxy for those who did not participate in genetic testing. Also, results for 25 year old men and women in 2017-2019 are extrapolated from a broader age range.*  *Combining this unique dataset with such a powerful polygenic predictor is the principal strength of our study. Although the GPS does not account for the effect of rare gene variants recently recovered by whole-genome sequencing [9], it is the first genetic instrument to provide meaningful predictive power by encompassing over 2 million common gene variants associated with obesity. Compared to the previous score [5], the increase in explained variance from 3% to 9% may appear small.*  Discussion, (Comparison with other studies), paragraph 2  *Compared to the British study, our study lacks statistical power in the younger age groups and could not replicate findings of an increasing weight gradient across polygenic score tenths from childhood to early adulthood [10]. Our dataset is however robust from age 25 to 75 years and does not affirm any clear age trends.* |
| Interpretation | 20 | Give a cautious overall interpretation of results considering objectives, limitations, multiplicity of analyses, results from similar studies, and other relevant evidence  Discussion, (Conclusion), paragraph 9  *The prevalence of obesity increased substantially from the mid-1980s and has stabilized to a new level over the last decade in the Norwegian population.* *While obesity is a highly heritable trait, [9] our study illustrates how it is still modifiable by the obesogenic exposure. Utilizing the substantial changes in our environment over time, we expose a growing inequality in risk for obesity and severe obesity between the genetically predisposed and lesser predisposed. The magnitude of our findings using the GPS is far greater than previously anticipated, holds true over time and is robust to confounding.*  Discussion, Implications and future research, clinical practice and public policy, paragraph 8  *Our findings suggest a genetic inequality in obesity during the obesity epidemic in Norway. This novel insight may help identify those susceptible to environmental change as a preventative measure.*  *Future research should focus on specific gene environment interactions that could help determine which preventative efforts are most effective. Regardless, secular trends have increased body weight for both genetically predisposed and genetically non-predisposed people. It is time for the global community to recognize and to address the determinants of ill-health that foster the unhealthy environment in which we live[7, 11].* |
| Generalisability | 21 | Discuss the generalisability (external validity) of the study results  Discussion, Generalizability of the findings, paragraph 7  *The effect of the obesogenic environment on population weight acts partly through enhancing the effect of our genes [5].* *The magnitude seems to relate to the obesogenic exposure in the macroenvironment, conceptualized as year of assessment in this study. In contrast to most countries [7, 12], the prevalence of obesity in Norway stabilized over the last decade. As a result, we observe a stabilizing gradient in weight and only a slight increased risk of obesity across polygenic score tenths. Although difficult to measure, this finding could indicate a consistent obesogenic exposure in the macroenvironment in recent years. However, in countries where obesity prevalence is still increasing, these weight gradient trends across genetic susceptibility would likely be different.* |
| Other information | | |
| Funding | 22 | Give the source of funding and the role of the funders for the present study and, if applicable, for the original study on which the present article is based  Funding: MB was funded by The Liaison Committee for Education, Research and Innovation in Central Norway with project number 90057601 and GÅV was funded by the Norwegian Research Council with grant number 250335. JHB was funded by the Norwegian Research Council with grant number 295989.  BB and BOÅ work in a research unit funded by Stiftelsen Kristian Gerhard Jebsen; Faculty of Medicine and Health Sciences, NTNU; The Liaison Committee for Education, Research and Innovation in Central Norway; and the Joint Research Committee between St. Olavs Hospital and the Faculty of Medicine and Health Sciences, NTNU; and the Medical Research Council Integrative Epidemiology Unit at the University of Bristol which is supported by the Medical Research Council and the University of Bristol. GDS works in the Medical Research Council Integrative Epidemiology Unit at the University of Bristol MC_UU_00011/1.  The funders had no role in study design, data collection and analysis, decision to publish, or preparation of the manuscript. |

*Give information separately for cases and controls in case-control studies and, if applicable, for exposed and unexposed groups in cohort and cross-sectional studies.

**Note:** An Explanation and Elaboration article discusses each checklist item and gives methodological background and published examples of transparent reporting. The STROBE checklist is best used in conjunction with this article (freely available on the Web sites of PLoS Medicine at http://www.plosmedicine.org/, Annals of Internal Medicine at http://www.annals.org/, and Epidemiology at http://www.epidem.com/). Information on the STROBE Initiative is available at [www.strobe-statement.org](http://www.strobe-statement.org/).

**References**
