# Supplementary material for: Genetic associations with temporal shifts in obesity and severe obesity during the obesity epidemic in Norway: A longitudinal population-based cohort (the HUNT Study)
Source: PLoS Med. 2020 Dec 14;17(12):e1003452. doi: 10.1371/journal.pmed.1003452 (PMC7735641; doi:10.1371/journal.pmed.1003452)
Supplement: Attachment — Submitted filename: Response to reviewers.docx [file pmed.1003452.s005.docx]

Response to Reviewers

Requests from the editors:

*One reviewer comments on the overlap between the analyses in this paper and those in a recent BMJ article, focusing on the effect of a 97-variant genetic risk score and obesity risk. The editors felt that these concerns did not invalidate the paper from further consideration in PLOS Medicine but it would be good to try to be more clear in the paper what is the value and novel contribution of the present genome-wide score.

The GPS is an impressive tool that directly assesses genetic propensities of individuals. When comparing the GPS with the GRS used in our previous study, we understand that the increase in explained variance from 2.7% to 9% may not seem impressive at first. However, on a population level, this translates to a considerable increased difference in BMI between people categorized as more or less predisposed to obesity. More importantly, the resulting difference in BMI is on a scale relevant to clinical practice. For further detail, please see the response to the third reviewer’s first question. By classifying genetic propensity more accurately, the GPS enables us to better investigate the interplay between genes and the obesogenic environment in the diseased ranges of BMI.

Hence, we believe that utilizing the GPS in analyses focusing on obesity and severe obesity is a valuable contribution to this line of work. Another important novelty of this article is that we validate the interplay between genes and the obesogenic environment with sibling design. Finally, we expand the dates of assessment to as recent as 2019 and capture the influence of both an increasing and stabilizing prevalence of obesity.

We revised the following text in the introduction:

*Utilizing the dramatic changes in our environment from 1966 to 2019, we now apply the vastly more powerful GPS to show that the same trends exist with the prevalence of obesity.*

*This is important as it reduces or eliminates bias from genetic assortment, population stratification, and dynastic effects. Hence, this study provides the most powerful approach to date to quantify and validate the interplay between our genes and the environment.*

Similarly, the following text was revised in the discussion: *For 35 year old men and women, the absolute increase in prevalence of obesity using the powerful GPS was 20 percentage points greater for the most genetically predisposed tenth compared with the least predisposed tenth.*

*Compared to the previous score,(1) the increase in explained variance from 3% to 9% may seem small at first. However, this translates to a substantially greater difference in BMI between the genetically predisposed and lesser predisposed and improves the classification of genetic risk considerably. Among today’s middle-aged adults, this difference amounts to a 13kg weight gradient across polygenic score tenths.(2)*

*In this study we validate our findings in between-sibling analyses, which demonstrate that neither assortative mating, dynastic effects, nor fine-scale population stratification issues generate the results we see.(3-5) However, we recognize that parents of participants in our cohort met before the start of the obesity epidemic in the mid-1980s and genotypic assortment for BMI might be a greater issue in the future.*

*Please include a brief note in the last sentence of the Abstract Methods and Findings section summarising any key limitation(s) of the study's methodology.

We added the following sentence to the Abstract Methods and Findings section:

*Key limitations include a slight differential survival to date of genetic testing and that we apply a contemporary genetic score to past time periods*.

*At this stage, we ask that you include a short, non-technical Author Summary of your research to make findings accessible to a wide audience that includes both scientists and non-scientists. The Author Summary should immediately follow the Abstract in your revised manuscript. This text is subject to editorial change and should be distinct from the scientific abstract. Please see our author guidelines for more information: <https://journals.plos.org/plosmedicine/s/revising-your-manuscript#loc-author-summary>

The author summary is included in the revised manuscript.

*Please clarify, ideally in the Methods section, if the analytical approach used in this paper corresponded to one laid out in a prospective protocol or analysis plan? Please state this (either way) early in the Methods section.

a) If a prospective analysis plan (from your funding proposal, IRB or other ethics committee submission, study protocol, or other planning document written before analyzing the data) was used in designing the study, please include the relevant prospectively written document with your revised manuscript as a Supporting Information file to be published alongside your study, and cite it in the Methods section. A legend for this file should be included at the end of your manuscript.

b) If no such document exists, please make sure that the Methods section transparently describes when analyses were planned, and when/why any data-driven changes to analyses took place.

N/A.

c) In either case, changes in the analysis-- including those made in response to peer review comments-- should be identified as such in the Methods section of the paper, with rationale.

We have added the prospective protocol and rationale for modifications in the Supporting information files and referred to this in the manuscript.

The Methods section now reads:

*Prior to commencing the analyses, we modified the prospective analysis plan slightly in agreement with all co-authors. The prospective protocol and reasoning for modifications are included in the supplementary files (S1, S2 Text).*

For data residing with a third party, authors are required to provide instructions with contact information for obtaining the data. PLOS journals do not allow statements supported by "data not shown" or "unpublished results." For such statements, authors must provide supporting data or cite public sources that include it.

The following instructions contain contact information for obtaining data used in this study.

Data sharing: Data from the HUNT Study used in research projects will when reasonably requested by others be made available upon request to the HUNT Data Access Committee ([hunt@medisin.ntnu.no](mailto:hunt@medisin.ntnu.no)). The HUNT data access information (available here: <http://www.ntnu.edu/hunt/data>) describes in detail the policy regarding data availability. The Norwegian Institute of Public Health will consider applications for data from the Tuberculosis screening program (<https://www.fhi.no/en/op/data-access-from-health-registries-health-studies-and-biobanks/>).

Comments from the reviewers:

Reviewer #1

**Genetic inequalities in obesity and severe obesity during the obesity epidemic: longitudinal findings from the HUNT Study verified by sibling design**

Maria Brandkvist, Johan Håkon Bjørngaard, Rønnaug Astri Ødegård, Ben Brumpton, George Davey Smith, Bjørn Olav Åsvold, Erik R Sund, Kirsti Kvaløy, Cristen J Willer, Gunnhild Åberge Vie.

**Reviewer’s Comments**

The submitted paper aims to assess whether the prevalence of obesity over the last 54 years has increased disproportionately for individuals who are genetically more susceptible to high BMI than for the least susceptible individuals, which would be an indication of gene-environment interactions affecting obesity. The paper improves over the previous literature by combining a large-scale longitudinal dataset (HUNT) with a recently developed genome-wide polygenic risk score to measure genetic susceptibility to BMI.

I was happy with the manuscript overall and believe that the quality of the conducted research is sufficient for publication. I only have a few comments, which relate mainly to the presentation of the authors’ work rather than the content.

In particular, there is one issue which I think is worth pointing out. The authors report the results of their analysis mainly in terms of the difference in the prevalence of obesity between the two groups (top and bottom decile in terms of genetic susceptibility to BMI). I am not sure if this measure is informative enough. My concern relates to the fact that obesity is defined by a cut-off point in the distribution of BMI (i.e. BMI above 30). As such, an overall increase in BMI across the population will translate into an increase in the prevalence of obesity in both groups, but the proportions of increase in each of the two groups will not necessarily be comparable.

The above plot illustrates this point. Here, I have generated a scenario in which there is no gene-environment interaction, but instead BMI increases by 4 points uniformly in the population between the beginning and the end of the study. The curves represent the distribution of BMI for the least predisposed tenth (left) and the most predisposed tenth (right). Black curves represent the distribution of BMI in the beginning of the study and red curves represent BMI in the end of the study. A visual inspection of the distribution of BMI suggests that the increase in BMI is the same between the two groups, hence there are no gene-environment interactions. However, in this toy scenario, the prevalence of obesity increases from 1.2% to 10.6% for the least predisposed tenth and from 6.7% to 30.9% for the most predisposed tenth.

To provide further evidence to support the presence of gene-environment interactions, I would suggest that the authors compare average BMI figures between the least predisposed and the most predisposed group, and report these numbers in addition to the prevalence of obesity in page 10 of the manuscript. I am encouraged by Figure S3 suggesting that the difference between average BMI in time is greater for the most predisposed group than for the least predisposed group – maybe it is worth including that figure in the main part, or plotting the distribution of BMI for each group in the beginning and the end of the study to aid comparison. I imagine there may also be some hypothesis test that can be performed to formally compare the differences between BMI from the beginning to the end of the study between the two groups, although I am not familiar with the relevant literature so I am not sure whether this is common or even necessary.

We agree that some important information is lost when dichotomizing BMI with obesity cut-offs and we are happy to move Figure S3 to the manuscript if this is requested by the editor. We chose to focus on obesity and severe obesity in this paper as these are commonly used clinical categories associated with comorbidity and increased health care needs. We feel that the BMI categories make it easier to conceptualize and convey the main message of this paper.

We compared the difference in BMI between the most and least genetically predisposed tenths over time in supplementary table S2 and we have now added the following sentence to the results section:

*For instance, the difference in mean BMI between the most and least genetically predisposed tenths was 1.45 kg/m^2^ (1.09 to 1.81 kg/m^2^) greater in recent years compared with the 1960s.*

We also generated distributional graphs for the entire study sample and by GPS categories. For the latter we used two age groups, 20-40 and 40-60 years, as the average age of participants differs across time. These are now presented as S3 Fig and S4 Fig.

Furthermore, we performed a new analysis with the natural logarithm as the outcome in order to approximate the percent change in BMI. We then used the post estimation command lincom to estimate the difference between the most and least predisposed tenths over time. These results are presented in the new S5 table. The estimates and confidence intervals presented in S2 and S5 tables suggest that the difference between the genetically predisposed and lesser predisposed people is greater in recent years and statistically significant.

A couple of additional, very minor comments:

1) It would be nice if the authors gave a brief summary of how the genome-wide polygenic score was developed in previous literature – for example, does it comprise of variants with p-values below a certain threshold? How does it handle variants in linkage disequilibrium?

The genome-wide polygenic score (GPS) for BMI is a quantitative measure of an individuals inherited susceptibility to obesity based on the combined effect of many common variants (minor allele frequency ≥ 1%). (2) Khera et al. created the GPS by first obtaining the average effects for each of the 2,100,302 genetic variants associated with BMI in the GWAS Study for BMI published by Locke et al. The summary statistics of this GWAS Study included up to 339,224 individuals and a linkage disequilibrium reference panel of 503 European samples from 1000 Genomes phase 3 version 5. (6) DNA polymorphisms with ambiguous strand (A/T or C/G) were removed from the GPS. Next, a recently developed computational algorithm (LDpred) was used to reweight each genetic variant according to the following; ‘the effect size and strength of statistical significance observed in the prior GWAS, the degree of correlation between a genetic variant and others nearby, a tuning parameter that denotes the proportion of variants with non-zero effect size.’ (2, 7) A range of 5 values was tested for the tuning parameter resulting in five scores. Each score was validated and subsequently, the best GPS was selected. The GPS was strongly associated with observed BMI in the validation dataset of 119, 951 middle-aged adult participants in the UK Biobank with a correlation of 0.292. Comparatively, a 141-variant score had a correlation of only 0.133when tested in the same validation dataset.

We have added the following explanation to the manuscript:

*Palindromic polymorphisms were excluded, however all available variants of sufficient quality were included regardless of the p-value of their association with BMI. Using a Bayesian approach, a posterior mean effect size was calculated for each variant encompassing the extent at which similarly associated variants correlated with eachother in a reference population.(2)*

2) When reporting the prevalence of obesity, please report one additional decimal figure. This would turn slightly awkward statements of the form “estimate 1%, 95% CI 1% to 1%” to more informative ones, e.g. “estimate 1.0%, 95% CI 0.7% to 1.3%”.

We chose to report the prevalence of obesity without an additional decimal for clarity and simplicity. However, we do recognize that the first statement is awkward and therefore we changed all estimates to one decimal point.

The abstract now reads:

*Among 35 year old men, the prevalence of obesity for the least predisposed tenth increased from 0.9% (95% confidence interval [CI] 0.6 to 1.2%) to 6.5% (95% CI 5.0 to 8.0%) while for the most predisposed tenth it increased from 14.2% (95% CI 12.6 to 15.7%) to 39.6% (95% CI 36.1 to 43.0%). Equivalently for women of the same age, the prevalence of obesity for the least predisposed tenth increased from 1.1% (95% CI 0.7 to1.5%) to 7.6% (95% CI 6.0 to 9.2%) while the most predisposed tenth increased from 15.4% (95% CI 13.7 to 17.2%) to 42.0% (95% CI 38.7 to 45.4%). Thus, for 35 year old men and women respectively, the absolute change in prevalence of obesity from 1966 to 2019 was 19.8 percentage points (95% CI 16.2 to 23.5) and 20.0 percentage points (95% CI 16.4 to 23.7) greater for the most predisposed tenth compared with the least predisposed tenth, defined using the genome-wide polygenic score for BMI. The corresponding absolute change in prevalence of severe obesity for men and women respectively, was 8.5 percentage points (95% CI 6.3 to 10.7) and 12.6 percentage points (95% CI 9.6 to 15.6) greater for the most predisposed tenth.*

The results section now reads:

*Among 35 year old men, the prevalence of obesity for the least predisposed tenth increased from 0.9% (95% confidence interval [CI] 0.6 to 1.2%) to 6.5% (95% CI 5.0 to 8.08%) while for the most predisposed tenth it increased from 14.2% (95% CI 12.6 to 15.7%) to 39.6% (95% CI 36.1 to 43.0%). Hence, the absolute change in prevalence of obesity was 19.8 percentage points (95% CI 16.2 to 23.5 percentage points) greater for the highly predisposed. Equivalently for women of the same age, the prevalence of obesity for the least predisposed tenth increased from 1.1% (95% CI 0.7 to1.5%) to 7.6% (95% CI 6.0 to 9.2%) while the most predisposed tenth increased from 15.4% (95% CI 13.7 to 17.2%) to 42.0% (95% CI 38.7 to 45.4%). The absolute change in prevalence of obesity among women was 20.0 percentage points (95% CI 16.4 to 23.7 percentage points) greater for the highly predisposed (Figure 3, S4, S5 Tables). A similar trend is evident for severe obesity (Figure 4, S4, S5 Tables); the corresponding absolute change in prevalence of severe obesity for men and women respectively, was 8.5 percentage points (95% CI 6.3 to 10.7 percentage points) and 12.6 percentage points (95% CI 9.6 to 15.6 percentage points) greater for the highly predisposed.*

Reviewer #2: Brandkvist et al. demonstrate an increase in effect of a genetic score for BMI over time. The study is well written and the methods appear adequate for the research question. I only have minor comments that I feel should be addressed before the manuscript is accepted for publication.

The main result, an increase in genetic effect over time, is interpreted as a consequence of an increasingly obesogenic environment. I agree that this is the likely causal factor. However the authors need to argue from reported environmental observations that are relevant for the cohort used in the study. I would appreciate references to reports that have measured changing habits in activity and food intake for Norwegians, preferably in the Nord-Trøndelag region or a comparable population such as other Scandinavians, for the time period in question.

Obesity increased in Norway starting between the mid-1980s and mid-1990s. In this time period, Norway experienced increased prosperity as a result of new working cultures, increased market consumption and likely changes personal transport and in eating patterns influenced by North America and the rest of Europe. (8-10) These are likely important factors that contributed to the rapid population-wide growth. The following references from Statistics Norway discuss the changing habits in food intake from the late 1950s to the late 1990s and a near doubling (1,000 000 to 1,786 000) of registered automobiles from the mid-1970s to the late 1990s. The latter indirectly reflects a change in activity level as driving an automobile is a sedentary mode of transport. Unfortunately, I was unable to find English translations to these references. The first reference is a table with changes in food consumption by product and year. A trend that worries dieticians is the decrease in the consumption of fish and potatoes as well as milk, porridge, bread and other grain products. Simultaneously, the consumption of meat products, non- alcoholic beverages (excluding cacao, coffee and tea) as well as beer and wines has increased exponentially. Interestingly, the consumption of sugar, butter, margarine and oils has reduced by more than half over the time period. However, these statistics do not account for the quantity of these products added to pre-processed foods and beverages. A comparable Swedish study shows a substantial increase in pre-processed foods from 1960 to 2010.(11)

While there has been an increased consumption in fruits and berries and a slight increase in vegetables, the availability these products have likely increased as well.

<https://www.ssb.no/a/histstat/aarbok/ht-0502-222.html>

<https://www.ssb.no/a/histstat/artikler/art-2001-02-22-01.html>

<https://www.ssb.no/transport-og-reiseliv/artikler-og-publikasjoner/bilen-ble-allemannseie-i-1960>

The following sentence was added to the discussion:

*From the mid 1980s, Norway experienced increased prosperity resulting in new working cultures, increased market consumption and automobile transport as well as an unfavorable change to dietary patterns.(12, 13)*

In addition, the phrasing in the discussion: "Our findings show an interplay between genes and the environment [..]", should be modified since environmental factors were not included in statistical models. Rather, they were inferred by assumption of changes in environment over time with no reference to measured or reported data on environmental factors.

The revised sentences in the discussion now reads:

*Despite being a very heritable trait, our study illustrates that body weight is modifiable proportionate to the presumed degree of the obesogenic exposure. Hence, our findings comply with an interplay between genes and the environment that is robust to family-level confounding using sibling design.*

The revised sentence in the conclusion now reads:

*While obesity is a highly heritable trait,(14) our study illustrates how it is still modifiable according to the presumed degree of the obesogenic exposure.*

Figures 3 and 4 are missing legends.

The legends for Figure 3 and 4 should now be included in the manuscript

Reviewer #3: Study by Brandkvist and colleagues:

1-The authors recently published a study investigating the same question with a 97 SNP-based polygenic score versus a genome-wide polygenic score here. As these scores respectively explain 2.7% and 9% of BMI variation in the population, we do not expect very different conclusions from the two studies. In that context, the results of the current study, while interesting, have a limited incremental value.

We understand this comment however we believe that utilizing the powerful GPS in analyses focusing on obesity and severe obesity is an invaluable contribution to this line of work. For more details, please see the answer provided to the editors’ first comment and the corresponding changes made to the text in the manuscript.

In further analyses, we calculated the correlation between the GPS and GRS to be 0.48. This implies that the GPS provides a substantial amount of new information compared with the GRS. The following tables compare the GPS to the GRS in our dataset. While there is a substantial overlap in degree of genetic predisposition to obesity according to both genetic scores, several individuals are categorized differently using the new GPS. Also, the difference in average BMI between the highest and lowest tenth of genetic predisposition has increased from 2.2 kg/m2 using the GRS to 4.0 kg/m2 using the new GPS. Thus, we have good reason to believe that the GPS improves the accuracy in classification of genetic predisposition substantially.

We would also like to emphasize the value of the sibling analyses performed in this study. While the possibility of bias from assortative mating was questioned in the previous article, this study is robust to such forms of confounding.

Table 1 Number of individuals in each decile of GPS and GRS comparatively

|  | GPS decile 0 | GPS decile 1 | GPS decile 2 | GPS decile 3 | GPS decile 4 | GPS decile 5 | GPS decile 6 | GPS decile 7 | GPS decile 8 | GPS decile 9 | GPS decile 10 |
| --- | --- | --- | --- | --- | --- | --- | --- | --- | --- | --- | --- |
| GRS decile 0 | 2,245 | 1,324 | 957 | 737 | 557 | 408 | 298 | 214 | 134 | 49 | 6,923 |
| GRS decile 1 | 1,313 | 1,167 | 1,014 | 838 | 714 | 615 | 485 | 406 | 240 | 120 | 6,912 |
| GRS decile 2 | 958 | 1,054 | 934 | 836 | 786 | 674 | 596 | 508 | 364 | 212 | 6,922 |
| GRS decile 3 | 715 | 843 | 859 | 864 | 846 | 771 | 663 | 569 | 504 | 286 | 6,920 |
| GRS decile 4 | 579 | 720 | 788 | 865 | 783 | 763 | 730 | 675 | 631 | 391 | 6,925 |
| GRS decile 5 | 420 | 612 | 659 | 735 | 790 | 805 | 877 | 763 | 716 | 549 | 6,926 |
| GRS decile 6 | 319 | 457 | 623 | 623 | 750 | 819 | 858 | 907 | 865 | 704 | 6,925 |
| GRS decile 7 | 204 | 376 | 507 | 617 | 703 | 769 | 884 | 968 | 947 | 948 | 6,923 |
| GRS decile 8 | 118 | 254 | 380 | 503 | 585 | 740 | 835 | 974 | 1,159 | 1,373 | 6,921 |
| GRS decile 9 | 51 | 113 | 201 | 303 | 406 | 557 | 696 | 937 | 1,360 | 2,290 | 6,914 |
| GRS Total | 6,922 | 6,920 | 6,922 | 6,921 | 6,920 | 6,921 | 6,922 | 6,921 | 6,920 | 6,922 | 69211 |

Table 2 Mean BMI for the lowest and highest decile of the GRS and GPS

| HUNT 2 Survey | Genetic score | Decile | Observations | Mean BMI | Std. Dev. | Min | Max |
| --- | --- | --- | --- | --- | --- | --- | --- |
|  | GRS | 0 | 5,637 | 25.3 | 3.7 | 14.8 | 46.9 |
|  | GPS | 0 | 5,614 | 24.4 | 3.3 | 15.8 | 44.4 |
|  | GRS | 9 | 5,599 | 27.5 | 4.4 | 16.4 | 53.3 |
|  | GPS | 9 | 5,484 | 28.4 | 4.6 | 15.8 | 53.3 |

2-The authors concluded that the increase in the prevalence of obesity during the 60 years follow-up was steeper among the genetically predisposed (low genetic risk: 35 year-old men: from 1% to 7%, 35 year-old women: from 1% to 8%; high genetic risk: 35 year-old men: from 14% to 40%; 35 year-old women: from 15% to 42%). This statement is true if we consider absolute changes in the prevalence of obesity. However, another way to look at the data is to consider that the prevalence of obesity was multiplied by 7-8 fold in the low genetic risk group versus 2.8-2.85 fold in the high genetic risk group during the last decades. With these data in mind, it looks like the obesogenic environment had a more detrimental impact on the development of obesity in the low genetic risk than the high genetic risk group ('environmental' obesity). Interpreting the data while considering the relative versus absolute changes in the prevalence of obesity is probably worth it.

We found it more meaningful to study the absolute increase rather than the relative increase in obesity over the time period. The relative change of obesity can be misleading as it is sensitive to differences initial obesity levels between the genetically predisposed and lesser predisposed people. The large relative increase in obesity for the least genetically predisposed people is explained by a very low prevalence of obesity in this group to start off with. This information is not as clinically relevant as comparing the absolute increase in obesity for the genetically predisposed to that of the lesser predisposed.

3-The authors focused on 35-year old populations as it maximizes the statistical power. It may be relevant to perform sensibility analyses to make sure that the results observed in this age group can be generalized to younger and older people in the HUNT study.

We have included the following sentence in the results section:

*Similar yet slightly smaller changes were found among other ages. (Figure 3, 4, S3 and S3 Table, S4 Table)*

4-'adults…children…are overweight or obese'. Please use people first language (adults…children…are having overweight or obesity').

Thank-you for this suggestion. We have made the following changes to the text:

*Approximately 60-80% of adults and 20-30% of children in the western world are having overweight or obesity*(15, 16)

*It is logical to assume that children of couples with obesity are likely to inherit a higher genetic risk for obesity and that genetic variance would amplify for each generation, in turn contributing to an increasing obesity prevalence.*

5-The authors mention a heritability estimate of 40% for BMI. Heritability can be measured using different designs (twins, families, general populations). Please mention a heritability range of 40-75% (see the recent meta-analysis of heritability studies by Strijecky et al. Obes Rev 2018).

Thank-you for your suggestion. We have included the recommended reference and changed the text so that it now reads:

*For a trait with 40-75 % cross-sectional heritability,(14, 17) body mass index (BMI) is still very modifiable by the obesogenic environment.(1, 18)*

6-'fat mass, as indicated by body mass index'. BMI is a poor surrogate of fat mass in non-obese populations. I suggest rephrasing the sentence. The authors can focus on BMI heritability estimates or can cite heritability studies for fat mass.

We agree that BMI is a poor surrogate of fat mass in non-obese populations and chose to remove *fat mass* from this sentence.

7-As BMI equals weight divided by height square, it is questionable to adjust BMI for height in my opinion (lack of independence between the covariate and the outcome).

This argument was discussed amongst the co-authors before commencing the analyses and we agree that adjusting BMI for height seems counterintuitive as the BMI formula incorporates height. Nevertheless, it is still debated whether the BMI formula accounts for weight differences amongst people of different heights appropriately.(19) This argument was the basis for the criticism we received for using BMI in the previous article. Although BMI is not especially correlated with height, we chose to adjust BMI for height in order to account for any effect of the six centimeters height increase in the population since the 1960s.(20) Based on this comment, we have also redone the analyses with BMI not adjusted for height to assert that the results are similar. These analyses are not included in the manuscript.

8-The authors did not mention any transformation for BMI (ex. logarithmic, ranked-based inverse normal transformation). These transformations are frequently used in literature to correct for the lack of normality of the BMI distribution.

For the main analysis we wished to use raw BMI as the results are easier to interpret and communicate. In the manuscript, we now refer to a supplementary table S5 with results based on the natural logarithm of BMI.
The methods section now reads:

*To approximate the relative rather than the absolute difference in BMI, we assessed the association between the GPS and the natural logarithm of BMI.*

The results section now reads:

*Using the natural logarithm of BMI as the outcome, we still found larger effect sizes in more recent years (S5 Table).*

9-The authors may describe in more detail what were the changes of weight / BMI across time both globally and in the genetic subgroups in the Results section.

Longitudinal development in BMI from the 1960s to 2008 was covered in our previous paper and despite having more recent BMI measurements (2017 to 2019), we chose not to redo this analysis. We report mean BMI for the different time periods in S2 Table however, we chose not to comment these numbers as they are not age adjusted.

We added a sentence to the results section that now reads:

*For instance, the difference in mean BMI between the most and least genetically predisposed tenths was 1.45 kg/m^2^ (1.09 to 1.81 kg/m^2^) greater in recent years compared with the 1960s.*

10-In the discussion, the authors may cite and comment the paper by Abadi et al. Am J Hum Genet 2017. This study suggests the existence of 'snowball' obesity genes that may provide a nice mechanistic explanation for the results of the HUNT study.

This is an interesting article and we thank-you for your suggestion. We have added the following text and reference to the discussion section:

*A possible explanation comes from another study suggesting that the effect of certain genetic variants associated with obesity increase in people with higher BMI and the enhanced genetic effects stem predominantly from gene by environment interactions. Interestingly, these findings apply for BMI but not for height.(21)*

-----------------------------------------------------------

1.	Brandkvist M, Bjorngaard JH, Odegard RA, Asvold BO, Sund ER, Vie GA. Quantifying the impact of genes on body mass index during the obesity epidemic: longitudinal findings from the HUNT Study. BMJ. 2019;366:l4067.

2.	Khera AV, Chaffin M, Wade KH, Zahid S, Brancale J, Xia R, et al. Polygenic Prediction of Weight and Obesity Trajectories from Birth to Adulthood. Cell. 2019;177(3):587-96.e9.

3.	Kong A, Thorleifsson G, Frigge ML, Vilhjalmsson BJ, Young AI, Thorgeirsson TE, et al. The nature of nurture: Effects of parental genotypes. Science. 2018;359(6374):424.

4.	Young AI, Benonisdottir S, Przeworski M, Kong A. Deconstructing the sources of genotype-phenotype associations in humans. Science. 2019;365(6460):1396-400.

5.	Brumpton B, Sanderson E, Heilbron K, Hartwig FP, Harrison S, Vie G, et al. Avoiding dynastic, assortative mating, and population stratification biases in Mendelian randomization through within-family analyses. Nature communications. 2020;11(1):3519.

6.	Locke AE, Kahali B, Berndt SI, Justice AE, Pers TH, Day FR, et al. Genetic studies of body mass index yield new insights for obesity biology. Nature. 2015;518(7538):197-206.

7.	Vilhjálmsson BJ, Yang J, Finucane HK, Gusev A, Lindström S, Ripke S, et al. Modeling Linkage Disequilibrium Increases Accuracy of Polygenic Risk Scores. Am J Hum Genet. 2015;97(4):576-92.

8.	Rodgers A, Woodward A, Swinburn B, Dietz WH. Prevalence trends tell us what did not precipitate the US obesity epidemic. The Lancet Public Health. 2018.

9.	Statistical Yearbook 1986: Central Bureau of Statistics of Norway; [Available from: <https://www.ssb.no/a/histstat/aarbok/1986.pdf> (2018-06-29).

10.	Statistical Yearbook 1987: Central Bureau of Statistics of Norway; [Available from: <https://www.ssb.no/a/histstat/aarbok/1987.pdf> (2018-06-29).

11.	Juul F, Hemmingsson E. Trends in consumption of ultra-processed foods and obesity in Sweden between 1960 and 2010. Public Health Nutr. 2015;18(17):3096-107.

12.	Monsrud J. Bilen ble allemannseie i 1960 1999 [Available from: <https://www.ssb.no/transport-og-reiseliv/artikler-og-publikasjoner/bilen-ble-allemannseie-i-1960>.

13.	Forbrukte mengder av mat- og drikkevarer pr. person pr. år, etter varegruppe. 1958-1998: Statistics Norway; [Available from: <https://www.ssb.no/a/histstat/aarbok/ht-0502-222.html>.

14.	Wainschtein P, Jain DP, Yengo L, Zheng Z, Cupples LA, Shadyab AH, et al. Recovery of trait heritability from whole genome sequence data. bioRxiv. 2019:588020.

15.	Finucane MM, Stevens GA, Cowan MJ, Danaei G, Lin JK, Paciorek CJ, et al. National, regional, and global trends in body-mass index since 1980: systematic analysis of health examination surveys and epidemiological studies with 960 country-years and 9.1 million participants. Lancet. 2011;377(9765):557-67.

16.	Skinner AC, Ravanbakht SN, Skelton JA, Perrin EM, Armstrong SC. Prevalence of Obesity and Severe Obesity in US Children, 1999-2016. Pediatrics. 2018;141(3):e20173459. Pediatrics. 2018;142(3).

17.	Stryjecki C, Alyass A, Meyre D. Ethnic and population differences in the genetic predisposition to human obesity. Obes Rev. 2018;19(1):62-80.

18.	Walter S, Mejia-Guevara I, Estrada K, Liu SY, Glymour MM. Association of a Genetic Risk Score With Body Mass Index Across Different Birth Cohorts. JAMA. 2016;316(1):63-9.

19.	Michels KB, Greenland S, Rosner BA. Does body mass index adequately capture the relation of body composition and body size to health outcomes? Am J Epidemiol. 1998;147(2):167-72.

20.	Roser M, Appel C, Ritchie H. Human Height 2013 [updated May 20192019-09-03]. Available from: <https://ourworldindata.org/human-height>.

21.	Abadi A, Alyass A, Robiou du Pont S, Bolker B, Singh P, Mohan V, et al. Penetrance of Polygenic Obesity Susceptibility Loci across the Body Mass Index Distribution. Am J Hum Genet. 2017;101(6):925-38.
