# Supplementary material for: Genetic associations with temporal shifts in obesity and severe obesity during the obesity epidemic in Norway: A longitudinal population-based cohort (the HUNT Study)
Source: PLoS Med. 2020 Dec 14;17(12):e1003452. doi: 10.1371/journal.pmed.1003452 (PMC7735641; doi:10.1371/journal.pmed.1003452)
Supplement: Attachment — Submitted filename: Final revision.docx [file pmed.1003452.s006.docx]

Requests from the editors:

1.Comments from the Academic Editor: Response to Reviewer 3: Please include tables 1 and 2 from your response to Reviewer 3’s comments in the text of the manuscript (comparing the performance of the earlier GRS versus the more recent polygenic risk score) as this is helpful to emphasizing the advance of this study compared to previous reports using genetic risk score.

Table 1 and Table 2 are now included in the text of the manuscript.

Table 1 Number of individuals in each decile of GPS and GRS comparatively

|  | GPS decile 0 | GPS decile 1 | GPS decile 2 | GPS decile 3 | GPS decile 4 | GPS decile 5 | GPS decile 6 | GPS decile 7 | GPS decile 8 | GPS decile 9 | GPS decile 10 |
| --- | --- | --- | --- | --- | --- | --- | --- | --- | --- | --- | --- |
| GRS decile 0 | 2,245 | 1,324 | 957 | 737 | 557 | 408 | 298 | 214 | 134 | 49 | 6,923 |
| GRS decile 1 | 1,313 | 1,167 | 1,014 | 838 | 714 | 615 | 485 | 406 | 240 | 120 | 6,912 |
| GRS decile 2 | 958 | 1,054 | 934 | 836 | 786 | 674 | 596 | 508 | 364 | 212 | 6,922 |
| GRS decile 3 | 715 | 843 | 859 | 864 | 846 | 771 | 663 | 569 | 504 | 286 | 6,920 |
| GRS decile 4 | 579 | 720 | 788 | 865 | 783 | 763 | 730 | 675 | 631 | 391 | 6,925 |
| GRS decile 5 | 420 | 612 | 659 | 735 | 790 | 805 | 877 | 763 | 716 | 549 | 6,926 |
| GRS decile 6 | 319 | 457 | 623 | 623 | 750 | 819 | 858 | 907 | 865 | 704 | 6,925 |
| GRS decile 7 | 204 | 376 | 507 | 617 | 703 | 769 | 884 | 968 | 947 | 948 | 6,923 |
| GRS decile 8 | 118 | 254 | 380 | 503 | 585 | 740 | 835 | 974 | 1,159 | 1,373 | 6,921 |
| GRS decile 9 | 51 | 113 | 201 | 303 | 406 | 557 | 696 | 937 | 1,360 | 2,290 | 6,914 |
| GRS Total | 6,922 | 6,920 | 6,922 | 6,921 | 6,920 | 6,921 | 6,922 | 6,921 | 6,920 | 6,922 | 69211 |

Table 2 Mean BMI for the lowest and highest decile of the GRS and GPS

| HUNT 2 Survey | Genetic score | Decile | Observations | Mean BMI | Std. Dev. | Min | Max |
| --- | --- | --- | --- | --- | --- | --- | --- |
|  | GRS | 0 | 5,637 | 25.3 | 3.7 | 14.8 | 46.9 |
|  | GPS | 0 | 5,614 | 24.4 | 3.3 | 15.8 | 44.4 |
|  | GRS | 9 | 5,599 | 27.5 | 4.4 | 16.4 | 53.3 |
|  | GPS | 9 | 5,484 | 28.4 | 4.6 | 15.8 | 53.3 |

2.Comments from the Academic Editor: Please make it clear in the text why a number of significantly younger individuals with lower BMI from YHUNT2 were included in a dataset of otherwise older individuals (see table S1 and S3).

Added to Results:

Hoping to fully utilize the wide age range of the dataset, we included BMI measurements of adolescents available from the 1960s and 2000-01. In the results however, we chose to focus on BMI measurements from adult participants as observations in the adolescent age range were limited (Table 3).

3. Response to Reviewer 3: Please add the additional sensitivity analysis done with BMI not adjusted for height to the supporting information files and refer to these in the Methods and Results (in response to Reviewer 3 comment 7).

The additional sensitivity analysis with BMI in models not adjusted for height is now included in the supporting information file and referred to as follows:

Methods:

In the statistical analyses, we adjusted BMI for height to account for any effect of the six centimeter height increase in the population since the 1960s.(21) As suggested by the reviewers, we repeated the analyses using BMI without adjustment for height and compared the results.

Results:

Additional analyses with BMI in models not adjusted for height asserted similar results (S6 Fig, S1 Table, S3 Table, S4 Table).

4. Competing Interests: Please add this statement to the manuscript's Competing Interests: "GDS is an Academic Editor on PLOS Medicine's editorial board.”

This sentence will be added to the Competing Interests section when submitting the revision.

5. Abstract: Methods and Findings: In the first sentence, a comma is missing from “67 110 individuals” (The comma is similarly missing from this number in the Author Summary, Methods, and Results sections).

The commas are now included.

6. Abstract: Methods and Findings: Please revise this sentence, as your study did not formally test a gene x environment interaction. We suggest: “The greater increase in BMI in genetically predisposed individuals over time was apparent after adjustment for family-level confounding using a sibling design.” or similar.

This sentence is rewritten as suggested.

7. Abstract: Methods and Findings: Please clarify what is meant by “slight differential survival to date of genetic testing” in the limitations statement.

The abstract now reads:

Genotyping required survival to and participation in the HUNT Study in the 1990s or 2000s (…)

Key limitations include a slightly lower survival to date of genetic testing for the older cohorts and that we apply a contemporary genetic score to past time periods.

8. Abstract: Methods and Findings: Please include both 95% CIs and p values for all results presented here.

All estimates in the abstract include 95% CIs. We have added p-values to the estimated differences between percentage points increase in prevalence among high compared to low genetically predisposed. We do not see that adding p-values to each estimated prevalence provides any further information for the reader, as testing the null-hypothesis of prevalence of obesity or severe obesity being zero does not make clinical sense. If the editor wishes us to do so, we can add p<0.001 to each estimated prevalence of obesity and severe obesity presented in the abstract.

9. Abstract: Conclusions: * Please address the study implications without overreaching what can be concluded from the data; For the Conclusions paragraph, we suggest the following revision; “In the context of increasingly obesogenic changes in our environment over six decades, our findings reveal a growing inequality in risk for obesity and severe obesity across genome-wide polygenic score tenths. Our results suggest that while obesity is a heritable trait at least in part, it is still modifiable by environmental factors. Whilst it may be possible to identify those most susceptible to environmental change, who thus have the most to gain from preventive measures, efforts to reverse the obesogenic environment will benefit the whole population and help resolve the obesity epidemic.”

This paragraph is rewritten as suggested.

10. Author Summary: Please structure the Author Summary using bullet points for each point, and restrict the number of points to 3 per section, and 1 sentence per bullet point, if possible. Please see our author guidelines for more information: <https://journals.plos.org/plosmedicine/s/revising-your-manuscript#loc-author-summary>

This section is rewritten as suggested. The last point is changed to:

-This study demonstrates that our environment has a major impact on top of - and interacting with - our inherited genetics, and enforces the need to identify and to address the specific factors that led to the population wide increase in obesity.

11. Author Summary: Why was this study done? In the 3rd point of this section, please remove the word "powerful" " In the 4th point of this section, please remove “vastly more powerful” as this is not informative.

We removed these words.

12.Author Summary: What did the researchers do and find?: Please revise to (or similar):

--We assessed the relationship between an obesogenic environment and the prevalence of obesity according to genetic predisposition over six decades in Norway, with increasing and stabilizing prevalence of obesity, using a genome-wide polygenic score for BMI and validating by sibling design.

--Using genetic data from 67,110 individuals aged 13 to 80 years with repeated height and weight measurements recorded between 1966 and 2019, we found that the prevalence of obesity differed between the participants with the highest and lowest genetic susceptibility to obesity for all ages at each decade, and the difference increased gradually from the 1960s to the 2000s to then stabilize over the last decade

..

--For example, for 35 year old men and women, the increase in prevalence of obesity was 20 percentage points greater for the most genetically predisposed tenth compared with the least predisposed tenth.

This section is revised as suggested.

13. Citations: For in-text citations, please use square brackets rather than parentheses and place citations before the punctuation, like this [1].

The citations are changed.

14. Introduction: First paragraph: Please replace the terms “developing” and “western world” by referring to high income countries rather than "developed" or "Western" countries, and to low or middle income countries rather than "developing countries"

These words are revised as suggested.

15. Introduction: Last paragraph: Please revise to “This is important as it mitigates bias…”

This paragraph is revised as suggested.

16. Introduction: Please conclude the Introduction with a clear description of the study question or hypothesis. Final sentence: Please revise to remove “the most powerful” as this is hyperbole. We suggest: “Hence, the goal of this study is to quantify and validate the interplay between our genes and the environment.” or similar.

This sentence is revised as suggested.

17.Methods: First paragraph: Please clarify the ages of the participants, as the first sentence states you included individuals aged 13 or older, and the last sentence indicates you did not include individuals aged younger than 14. (Please also be sure this is consistent throughout the manuscript)

We revised the following sentence to clarify this point.

From the tuberculosis screening data, we limited height and weight data to the time interval with most observations from 1966-69 and excluded participants younger than 14 years as they were not targets for total population surveillance.

18. Methods: Ethical approval: Please note the nature of informed consent (e.g. written informed consent).

We revised this section to clarify the nature of informed consent.

All adult participants gave written informed consent before taking part in the HUNT Study. This consent included linkage to other registries. Parents gave written informed consent for adolescents taking part in the Young HUNT Study.

19. Methods: Page 10: Please provide additional details on how the sibling analyses were done and how this strengthens the study.

This section is rewritten as follows:

Since the transmission of alleles from parent to offspring is random, the siblings have equal likelihood of inheriting any given genetic variant. To assess whether assortative mating, dynastic effects or population stratification influenced the results, we analysed the association of the GPS with height-adjusted BMI as well as with the prevalence of obesity within and between siblings. The sibships’ GPS average and each sibling’s deviation from the group GPS average were calculated and included as independent variables in the regression models with individuals clustered in sibships. We used maximum likelihood random effects regression models for height adjusted BMI and generalized estimated equation models for obesity and severe obesity. We adjusted for age with splines as previously described, and included interaction terms between sex and each of the aforementioned measures of genetic predisposition. The within sibship coefficient is an estimate for differentially genetically exposed siblings. Between sibship coefficients exceeding the within sibship coefficients would indicate confounding at the sibship level.

20. Methods: Statistical analysis: Please specify the significance level used to determine whether there were differences in your results (eg, P<0.05, two-sided) as well as the statistical test used to derive a p value.

The following sentence is added to the Statistical analysis:

We report p-values from regression models based on Wald tests and marginal differences based on the delta method, both two-sided and with significance level at 0.05.

21. Results: Please present the findings quantified with 95% CIs and p values to reflect statistical differences of comparisons referred to in the text. Specifically, please provide quantitative results, 95% CIs and p values for the increase in BMI/prevalence of obesity over time overall and for genetically predisposed vs. not predisposed groups. Please provide both the height adjusted and unadjusted analyses.

The estimates with confidence intervals and p values are now presented in Table 4, Table 5, Table 6, Table 7, S1Table, S2 Table, S3 Table and S4 Table.

22. Results: Please present the results of sibling analyses with 95%CIs and p values “We found comparable associations between polygenic risk score and BMI as well as obesity within and between sibling groups with little evidence of bias from assortative mating, population stratification or dynastic effects (Fig 2, S6 Fig)”

We have added this information in S5 Table and S6 Table.

23. Results: Page 12: Please provide the statistical results to substantiate this sentence, or change this to “observed to be steeper” or “apparently steeper” to indicate that this was not statistically tested. “The increase in prevalence of obesity and severe obesity was steeper among the genetically predisposed over the time period (Fig 3, Fig 4).”

This sentence was revised as suggested.

The increase in prevalence of obesity and severe obesity was observed to be steeper among the genetically predisposed over the time period (Fig 3, Fig 4).

24. Results Page 12-13: Please provide p values in addition to 95% CIs for absolute changes in obesity prevalence.

We have provided p values in addition to 95% CIs as suggested.

25. Results: Page 12: Please remove the terms “dramatically” and “substantially” from the following sentences. If statistical significance is meant, please replace with “significantly” where appropriate. “From relative stability in the 1960s to 1980s, the weight for both the genetically predisposed and nonpredisposed increased dramatically from the mid-1980s to the 2000s and then stabilized to a higher level over the past decade. Height-adjusted BMI differed substantially across polygenic score tenths for all ages and at each decade, and the difference varied proportional to the changes in population weight (S5 Fig, S2 Table).”

This sentence is revised as follows:

From relative stability in the 1960s to 1980s, the weight for both the genetically predisposed and non-predisposed increased dramatically from the mid-1980s to the 2000s and then stabilized to a higher level over the past decade. At different ages and decades, estimated height-adjusted BMI differed by 2.5 to 5 BMI units from top to bottom polygenic score tenths (Table 4). While population weight increased for both genetically predisposed and lesser predisposed people over time (S2 Table), the BMI increased more for the genetically predisposed (Table 5, S5 Fig).

26. Discussion: Please present and organize the Discussion as follows: a short, clear summary of the article's findings; what the study adds to existing research and where and why the results may differ from previous research; strengths and limitations of the study; implications and next steps for research, clinical practice, and/or public policy; one-paragraph conclusion.

The discussion is reorganized to fit the above format.

27. Discussion: Please revise the first sentence to: In this study, we observed that, in transitioning to an obesogenic environment from the 1960s to the late-2010s, the prevalence of obesity and severe obesity increased dramatically for the genetically predisposed yet remained relatively unchanged for the least genetically predisposed.

This sentence is revised as suggested.

28.Discussion: Page 14: Please remove “the most powerful” from this sentence as it is hyperbole. “Combining this unique dataset with the most powerful polygenic predictor to date is the principal strength of our study.” Please also remove other instances of hyperbole.

These sentences are revised to remove instances of hyperbole.

29. Discussion: please either remove or clarify this sentence, as the meaning is unclear in the context of your results: “Despite being a very heritable trait, our study illustrates that body weight is modifiable proportionate to the presumed degree of the obesogenic exposure.”

This sentence is clarified as follows. I believe this to be an important message in the paper however I accept that it be removed if preferred by the editor.

Conceptualizing year of assessment as a broad indicator of the obesogenic environment, our study illustrates that despite being a very heritable trait, body weight seems modifiable by obesogenic exposure.

30. Discussion: please either remove or clarify these sentences, as it isn’t clear that your results support this conclusion: “The effect of the obesogenic environment on population weight acts partly through enhancing the effect of our genes. The magnitude seems to relate directly with degree of obesogenic exposure in the macroenvironment.”

We clarify by adding a reference to the first sentence and revising the second sentence as follows. I accept that these sentences be removed if preferred by the editor.

The magnitude seems to relate to the obesogenic exposure in the macroenvironment, conceptualized as year of assessment in this study.

We also revised a similar sentence in the conclusion.

While obesity is a highly heritable trait,(6) our study illustrates how it is still modifiable by the obesogenic exposure.

31. References: Please use the "Vancouver" style for reference formatting, and see our website for other reference guidelines <https://journals.plos.org/plosmedicine/s/submission-guidelines#loc-references>

In particular, please check the formatting of ref. #5 for missing information, and please provide English translations for 27 and 28.

Reference 5, 26 and 27 are revised.

32. Checklist: Thank you for including the completed STROBE checklist as Supporting Information. Please revise the checklist, please use section and paragraph numbers (e.g. “Methods, paragraph 1”), rather than page numbers to refer to locations in the text.

Please add the following statement, or similar, to the Methods: "This study is reported as per the Strengthening the Reporting of Observational Studies in Epidemiology (STROBE) guideline (S1 Checklist)."

We have revised the STROBE checklist and revised the above sentence in the manuscript.

33. Figure 2: Please provide p values to accompany the ORs reported. Please note in the legend what is represented by each of the 5 panels.

We have added p values for the within and between estimates as well as for the difference between these estimates. The legend is revised as follows:

**Fig 2. Estimated odds ratio (with 95% confidence interval) for obesity per standard deviation higher GPS for BMI within and between siblings by year.** Based on 29,585 individuals composing 11,857 sibling groups within participants in the HUNT Study.

34. Figures 3 and 4: Please provide X axis labels. Please revise the Y axis label to read “Obesity/Severe Obesity Prevalance” or similar. Please use (and explain in the figure legend) different markers or colors to indicate the points and 95% CIs corresponding to the highest and lowest propensity scores.

We have provided X axis labels and revised the Y axis. We explain the difference markers in the legends that now read:

**Fig 3. Estimated prevalence of obesity by top and bottom tenth of genome-wide polygenic score.** Estimated prevalence (%, with 95% confidence interval) of obesity (BMI ≥ 30 kg/m^2^) by top (most susceptible, circle) and bottom tenth (least susceptible, x) of genome-wide polygenic score by age and time point for 31,717 men and 35,393 women who participated in the HUNT Study, Norway. *Youngest observed age in 2017-19 was 28.6 years.

**Fig 4.** **Estimated prevalence of severe obesity by top and bottom tenth of genome-wide polygenic score.** Estimated prevalence (%, with 95% confidence interval) of severe obesity (BMI ≥ 35 kg/m^2^) by top (most susceptible, circle) and bottom tenth (least susceptible, x) of genome-wide polygenic score by age and time point for 31,717 men and 35,393 women who participated in the HUNT Study, Norway *Youngest observed age in 2017-19 was 28.6 years.

35. S2 Figure: Please provide Y axis labels.

This graph is revised as suggested.

36. S3 Figure: Is there a chance some of the lines are out of order? The trend of increasing BMI over time doesn’t fit with this plot of BMI distribution for the entire study sample by time of measurement, in particular the 2000-2001 line seems out of place (if this is due to the fact that this was the year when only adolescents participated, please make note of this in the legend).

We have added information about the age range of participants at each time point in legend.

37. S5 Figure: Please use different colors or different marks (and explain in the legend) to indicate the top vs. bottom score data. In the legend, please revise 28,6 to 28.6.

We have revised as suggested and added a comparable figure for BMI (new S6 Fig). The legends of S5 Fig and S6 Fig now read:

S5 Fig Estimated height-adjusted BMI (with 95% confidence interval) by top (most susceptible, circle) and bottom tenth (least susceptible, x) of genome-wide polygenic score by age and time point for 31,717 men and 35,393 women who participated in the HUNT Study, Norway. *Youngest observed age in 2017-19 was 28.6 years.

S6 Fig Estimated BMI (with 95% confidence interval) by top (most susceptible, circle) and bottom tenth (least susceptible, x) of genome-wide polygenic score by age and time point for 31,717 men and 35,393 women who participated in the HUNT Study, Norway. *Youngest observed age in 2017-19 was 28.6 years.

38. S6 Figure: Please indicate in the legend the purpose of the different panels, and please include p values along with the 95% CIs. Please clarify if the X axis is “height adjusted BMI”

The figure (now S7 Fig) and the legend are revised as suggested.

39. S7, S8, and S9 Figures: Please use different colors or different marks (and explain in the legend) to indicate the top vs. bottom score data. Please provide X axis labels for all plots.

These figures and legends are revised as suggested. Please note that they are now S8 Fig, S9 Fig and S10 Fig.

40. Supporting Information Files: The supplemental information files S1 Table and S2 Table. S3 Table, S4 Table are central to the understanding of the paper. Please incorporate these into the main paper.

These tables are now incorporated in the main manuscript as Table 3, Table 5, Table 6 and Table 7.

41. S2Table: Please define abbreviations for SD and BMI in the legend.

These abbreviations are defined in Table 3.

42. S2 Table: Please also provide the p values associated with these differences. Please clarify if “BMI” represents the difference value, or another measure and provide units if applicable.

We made changes as suggested above and included this table as Table 5.

43. S3 Table: Please provide p values for these differences. Please clarify what is presented in the columns “Men” and “Women”

We made changes as suggested.

44. S4 Table: Please provide p values for these differences. Please make it more clear what is presented in the columns “Men” and “Women”

We made changes as suggested.

45. S5 Table: Please provide p values for these differences. Please indicate what is presented in column “BMI” and how this differs from the presentation of data in Table S2.

We made changes as suggested.

Comments from the reviewers:

Reviewer #1: I am satisfied with the manuscript and the edits the authors have made to address my previous comments. I therefore recommend that their work is accepted for publication.
